# Supplementary figures and images for: Comparative transcriptomics reveal a novel tardigrade-specific DNA-binding protein induced in response to ionizing radiation
Source: eLife. 2024 Jul 9;13:RP92621. doi: 10.7554/eLife.92621 (PMC11233135; doi:10.7554/eLife.92621)

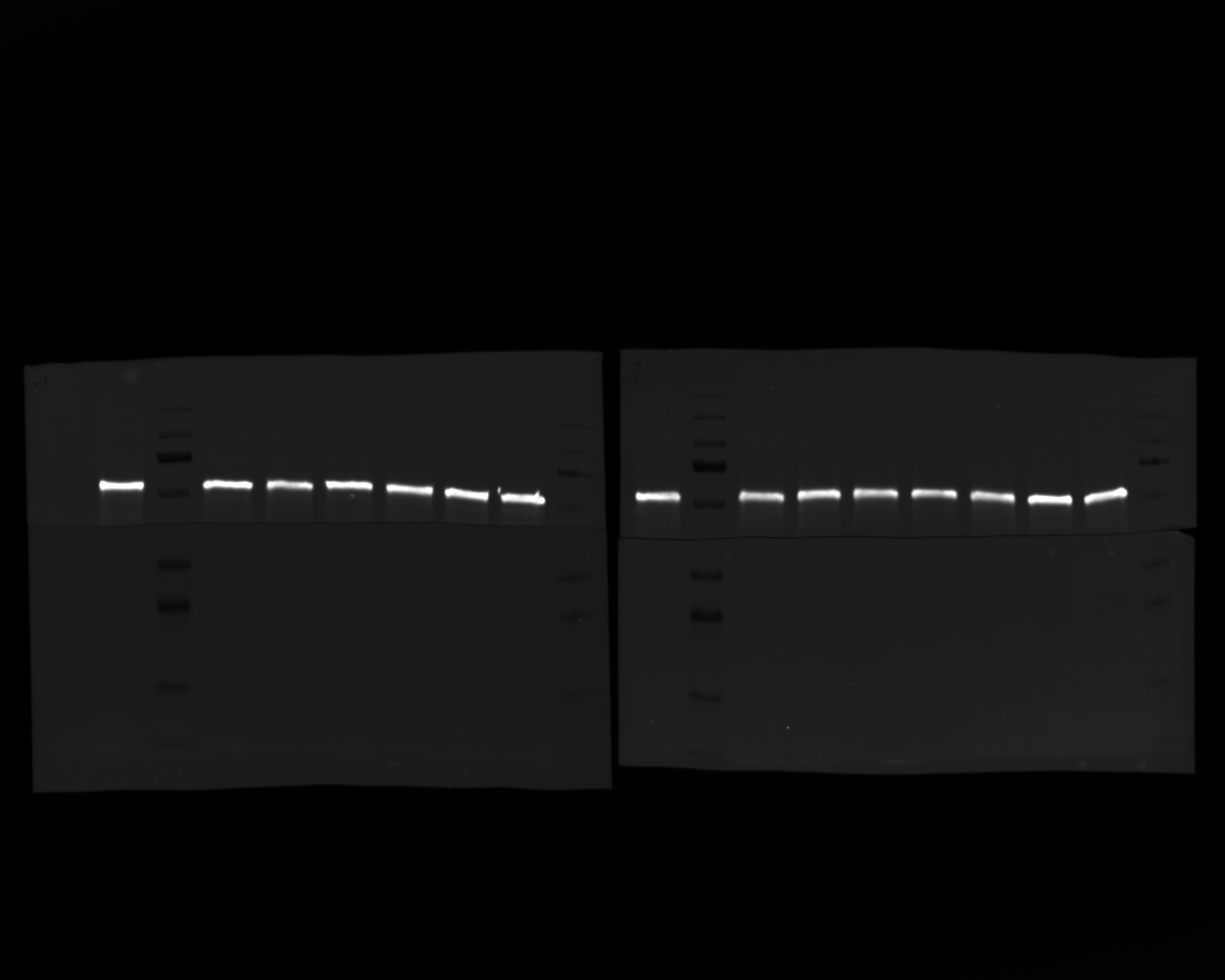

Supplement: Figure 1—source data 1. [file elife-92621-fig1-data1.zip › FIgure 1- source data raw data 16bits tifs/cin100-1000gy mb1-3 gh2ax-xrcc5 2021-09-15 15h45m09s Tubulin (StarBright B520).tif]

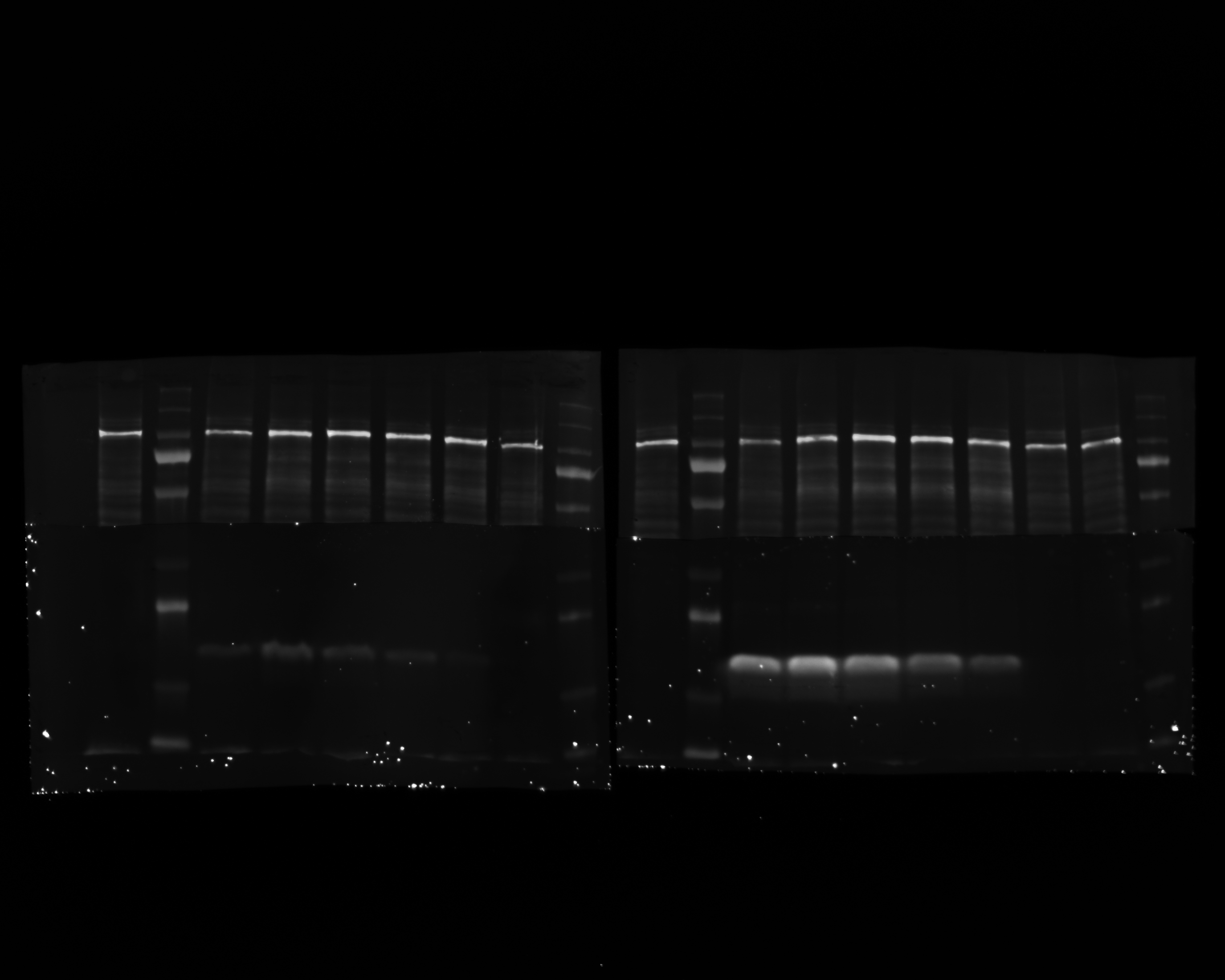

Supplement: Figure 1—source data 1. [file elife-92621-fig1-data1.zip › FIgure 1- source data raw data 16bits tifs/cin100-1000gy mb1-3 gh2ax-xrcc5 2021-09-15 15h45m09s up Xrcc5 down phosphoH2AX (StarBright B700).tif]

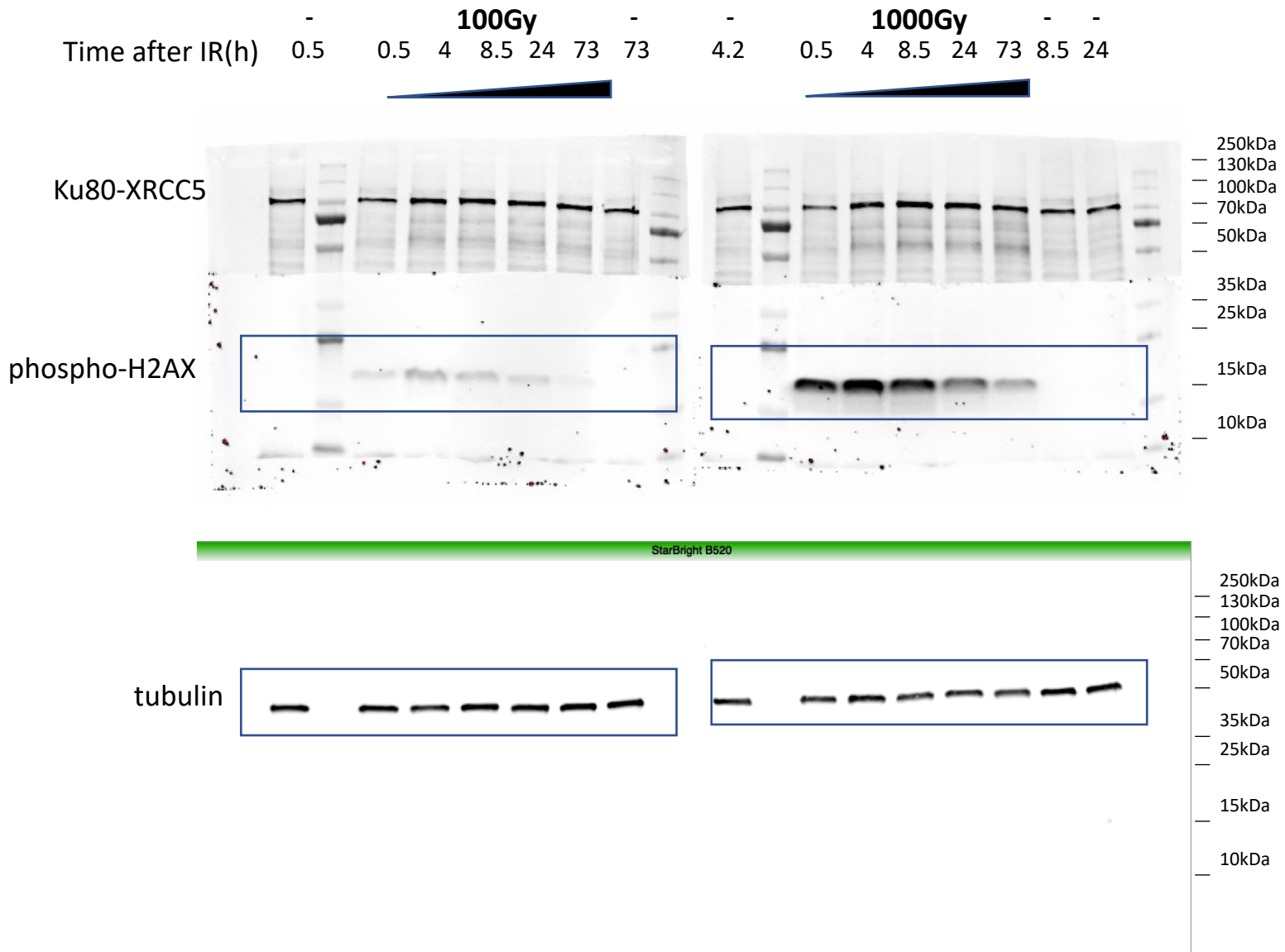

Supplement: Figure 1—source data 2. [file elife-92621-fig1-data2.pdf]

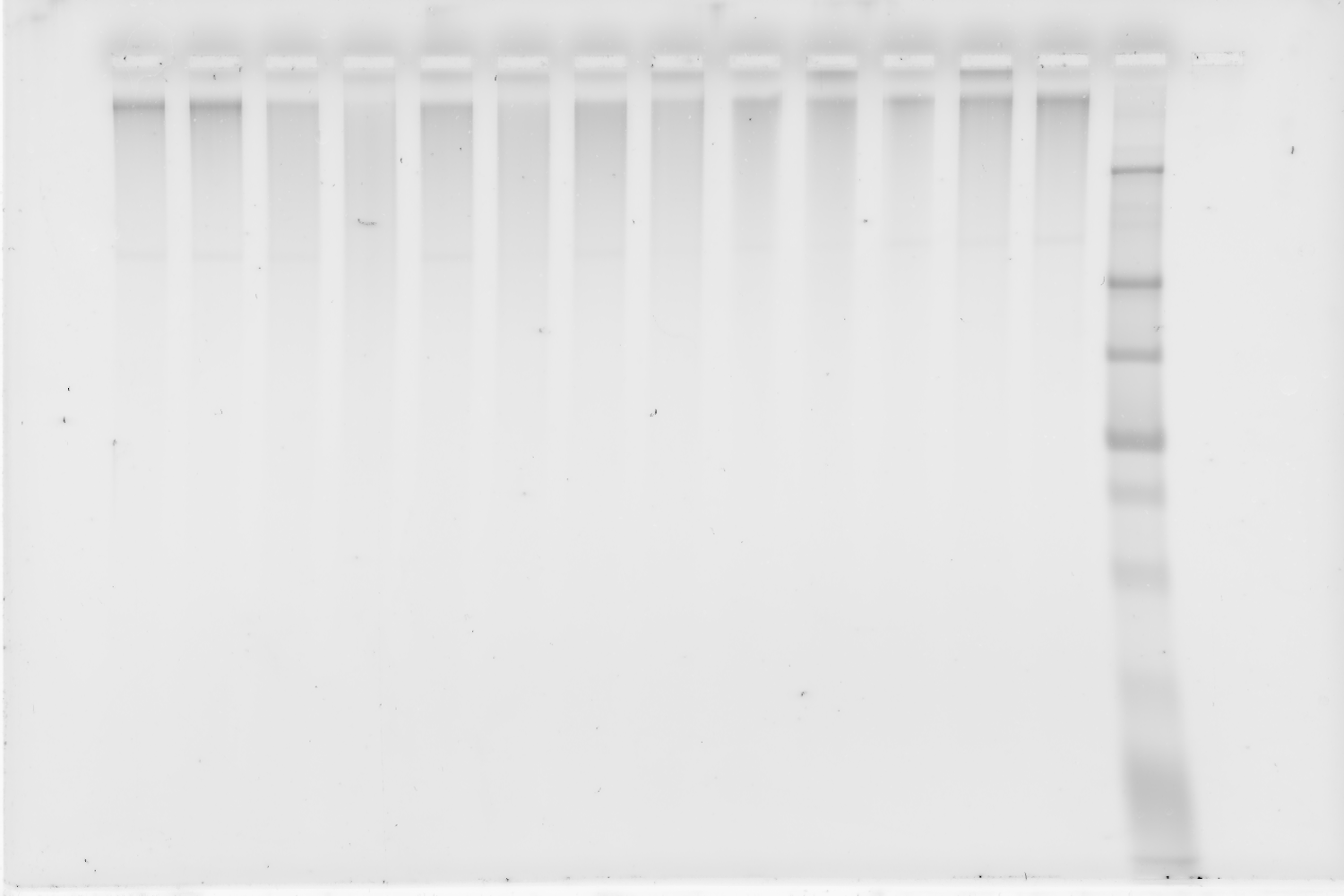

Supplement: Figure 1—source data 3. [file elife-92621-fig1-data3.zip › Figure 1 source data 3.tif]

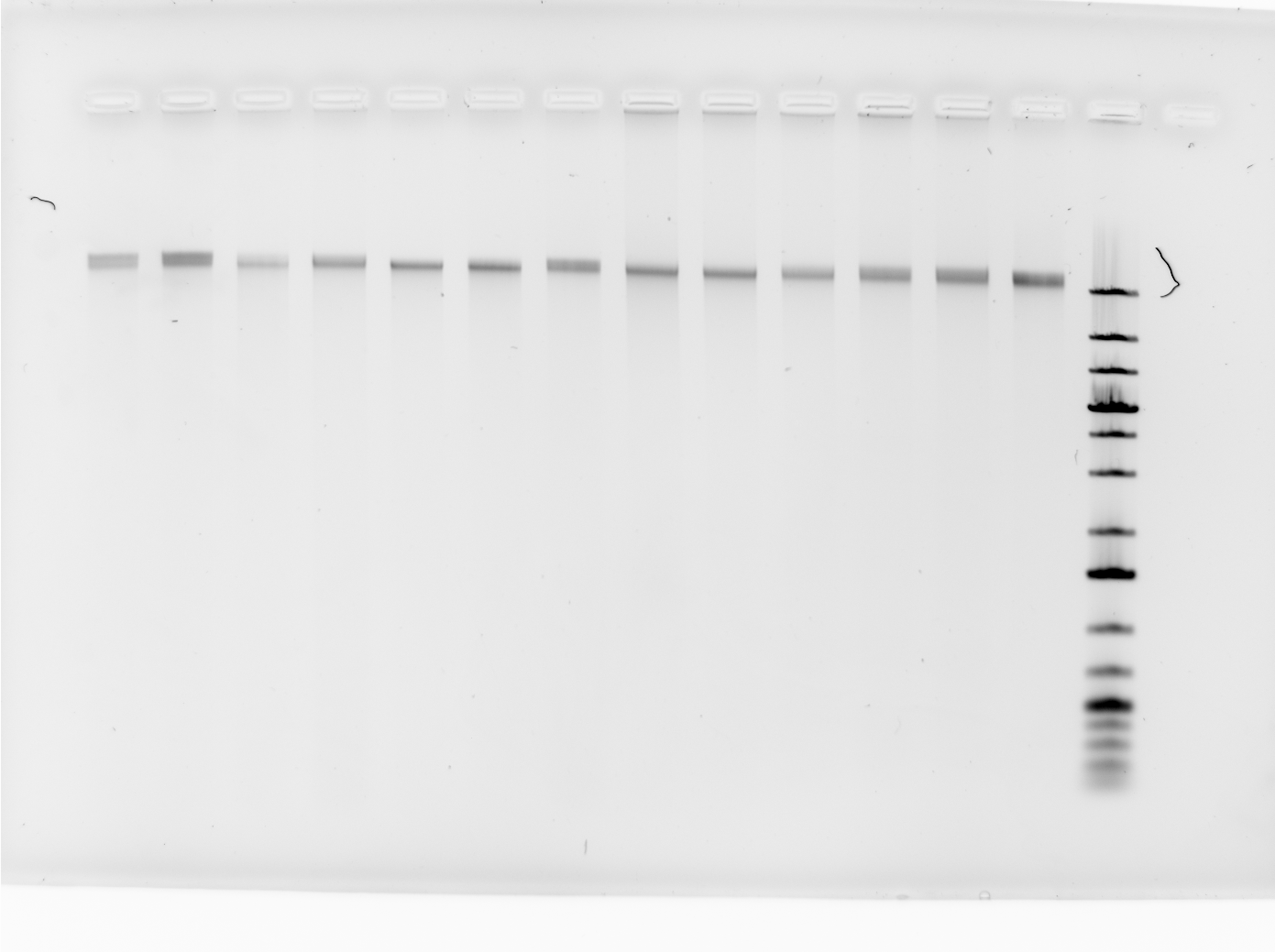

Supplement: Figure 1—figure supplement 3—source data 1. [file elife-92621-fig1-figsupp3-data1.zip › Figure 1 figure supplement 3 - source data 1.tif]

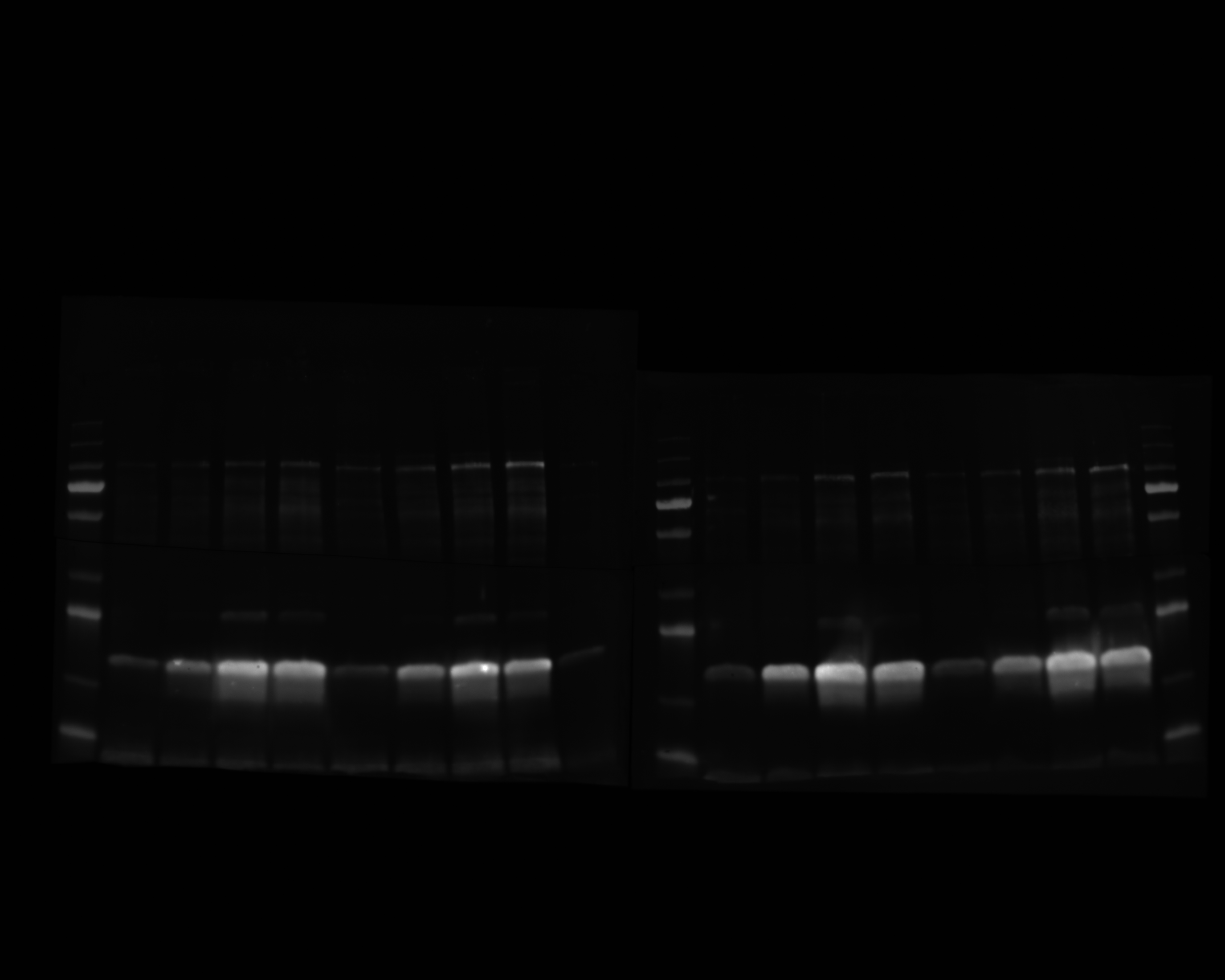

Supplement: Figure 3—source data 1. [file elife-92621-fig3-data1.zip › Figure 3- source data 1 - raw data 16bits tifs/prote-n2bleo-ir-mb1-4h2ax-xrcc5 2021-10-14 15h32m37s up xrcc5 down H2AXtotal (StarBright B700).tif]

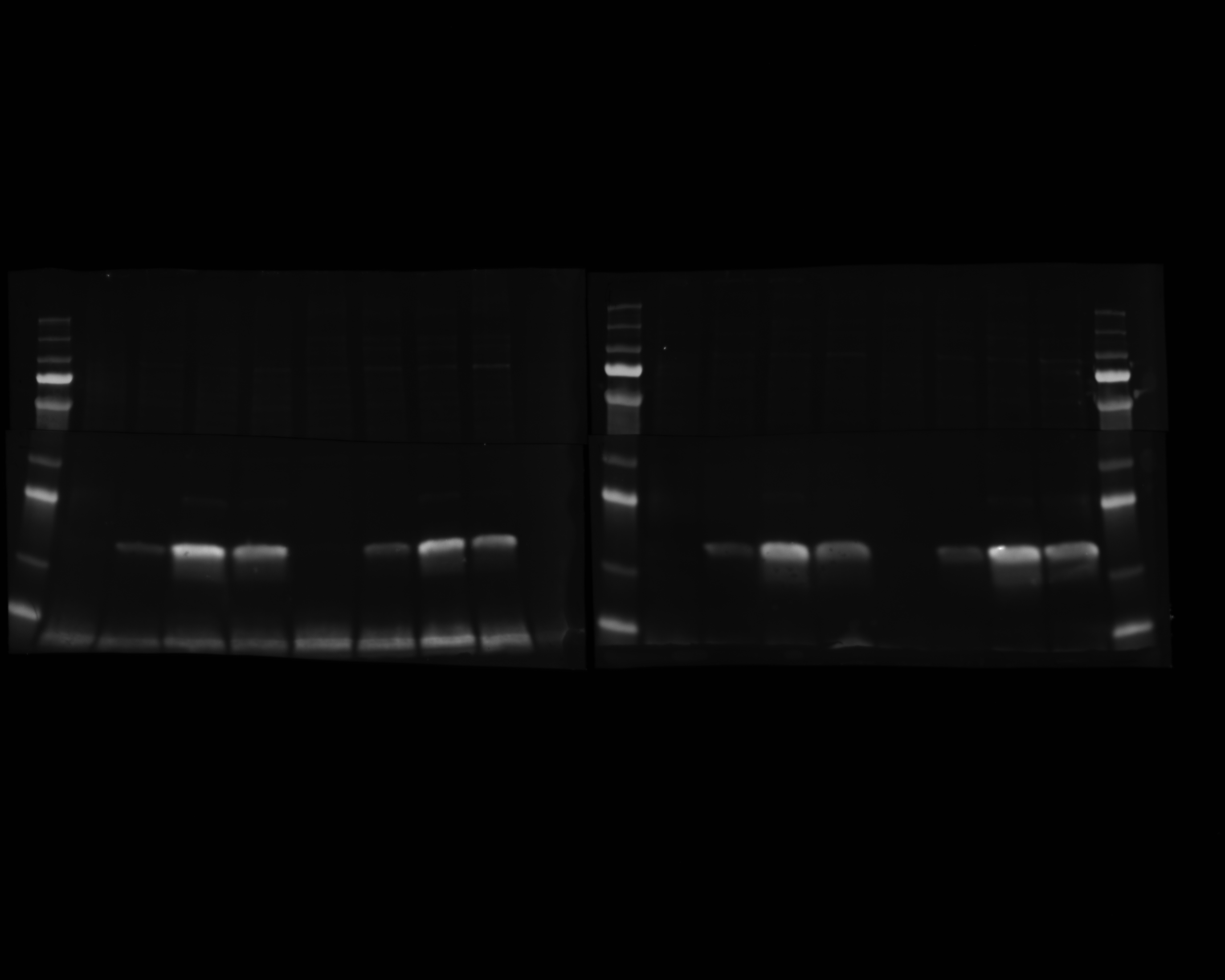

Supplement: Figure 3—source data 1. [file elife-92621-fig3-data1.zip › Figure 3- source data 1 - raw data 16bits tifs/prote-n2bleo-ir-mb2-5gh2ax-xrcc6 2021-10-14 15h24m25s up xrcc6 down phosphoH2AX (StarBright B700).tif]

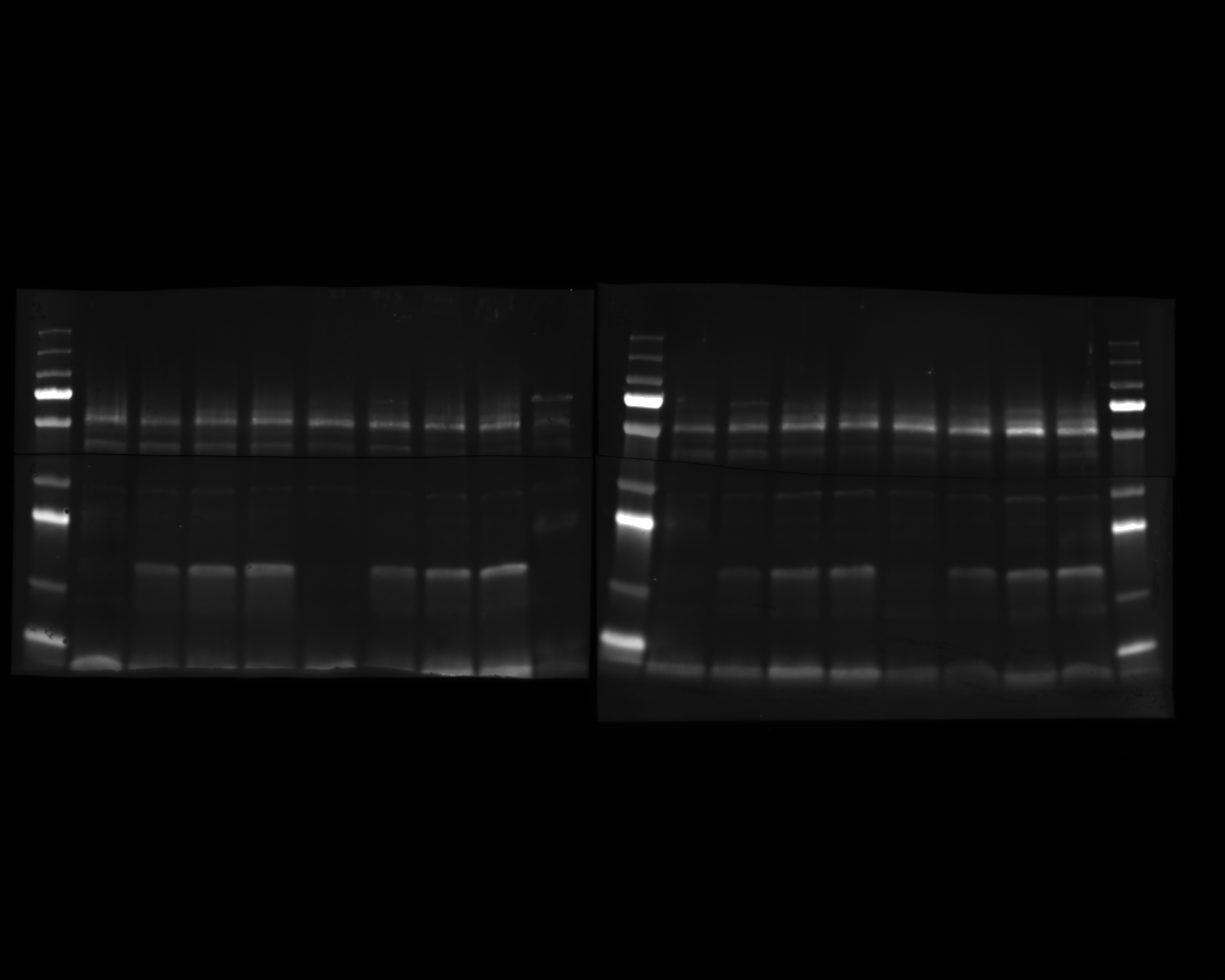

Supplement: Figure 3—source data 1. [file elife-92621-fig3-data1.zip › Figure 3- source data 1 - raw data 16bits tifs/prote-n2bleo-ir-mb3-6-TDR1-dsup 2021-10-14 15h38m08s up Dsup down HeTDR1 (StarBright B700).tif]

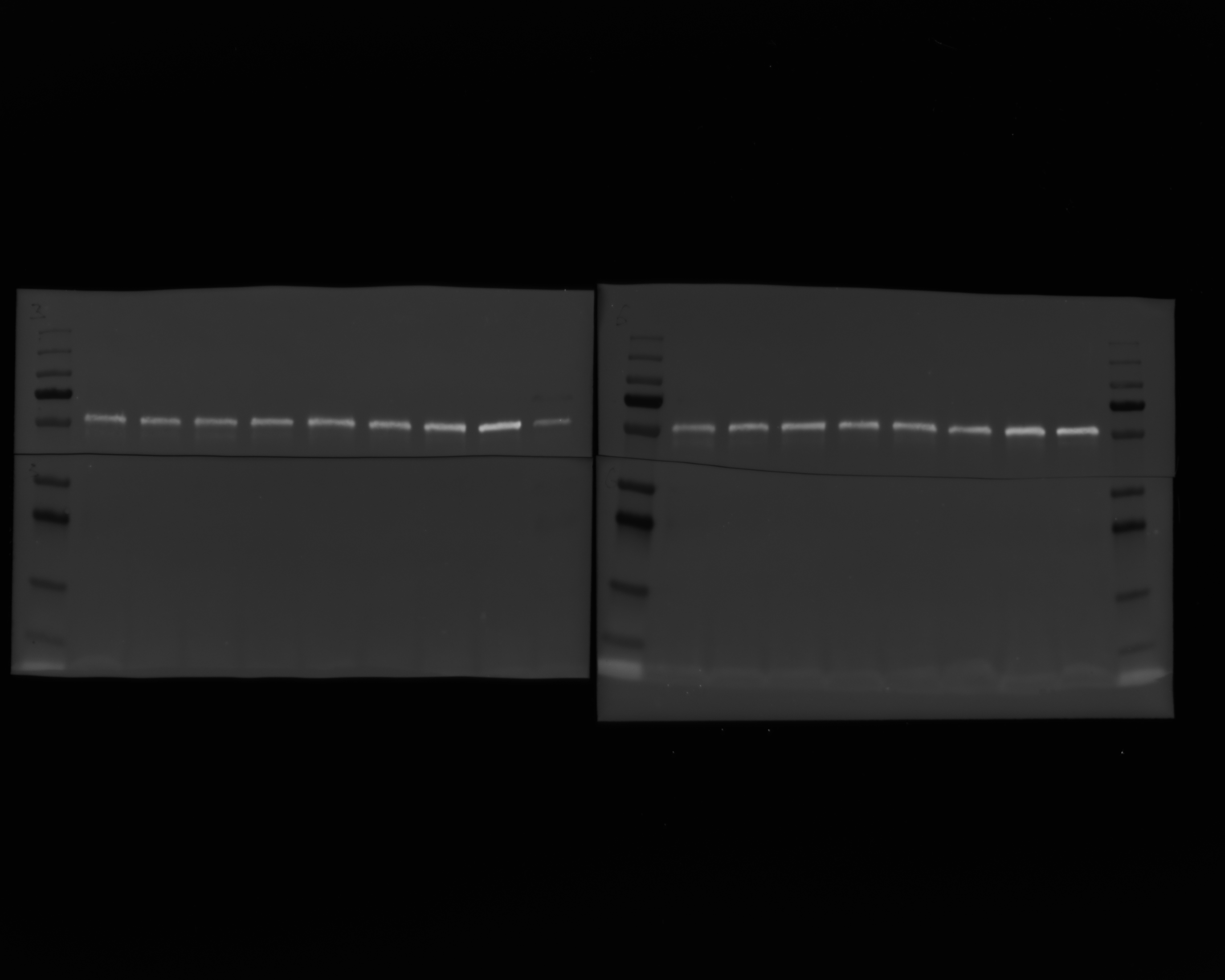

Supplement: Figure 3—source data 1. [file elife-92621-fig3-data1.zip › Figure 3- source data 1 - raw data 16bits tifs/prote-n2bleo-ir-mb3-6-TDR1-dsup 2021-10-14 15h38m08s Tubulin (StarBright B520).tif]

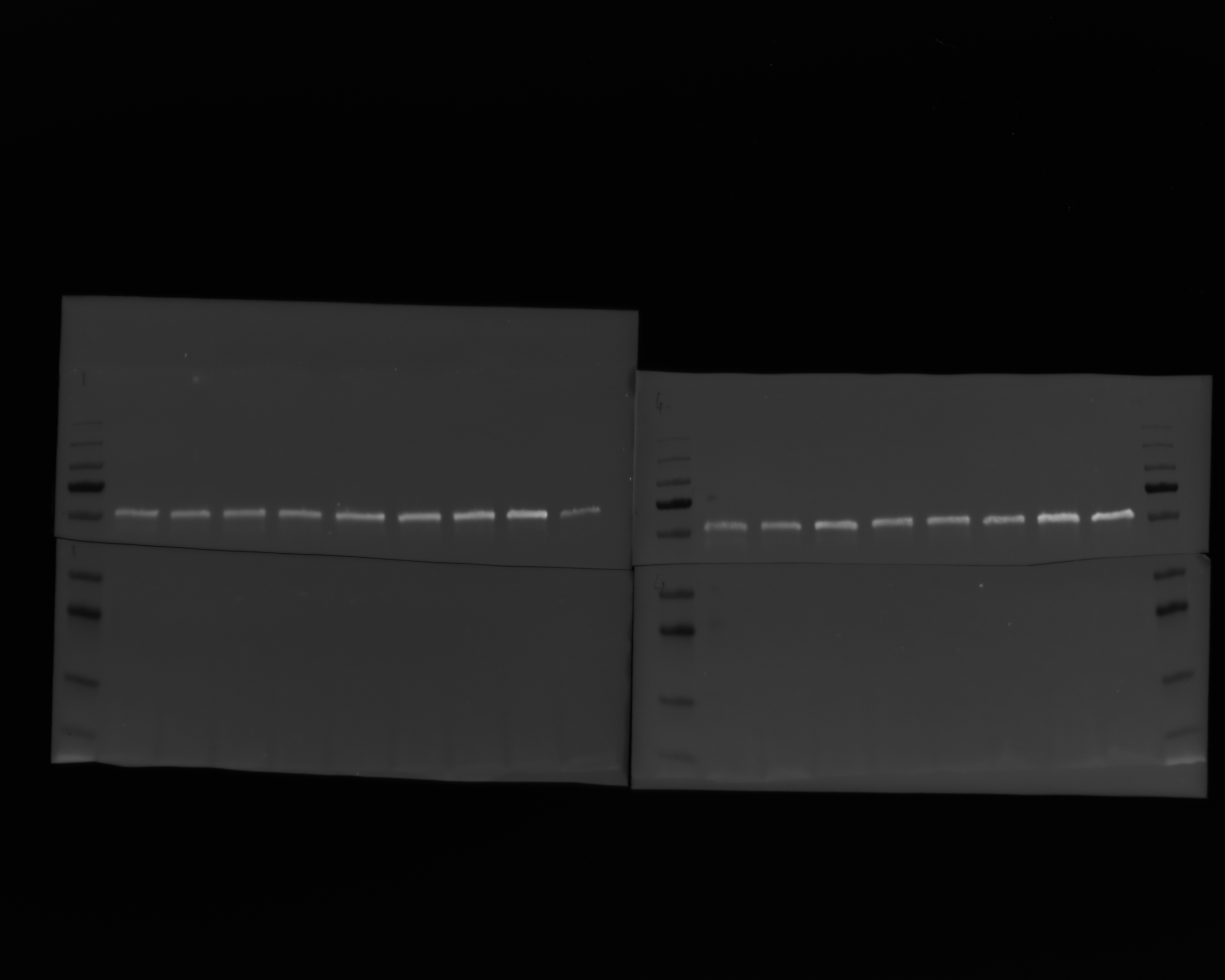

Supplement: Figure 3—source data 1. [file elife-92621-fig3-data1.zip › Figure 3- source data 1 - raw data 16bits tifs/prote-n2bleo-ir-mb1-4h2ax-xrcc5 2021-10-14 15h32m37s Tubulin (StarBright B520).tif]

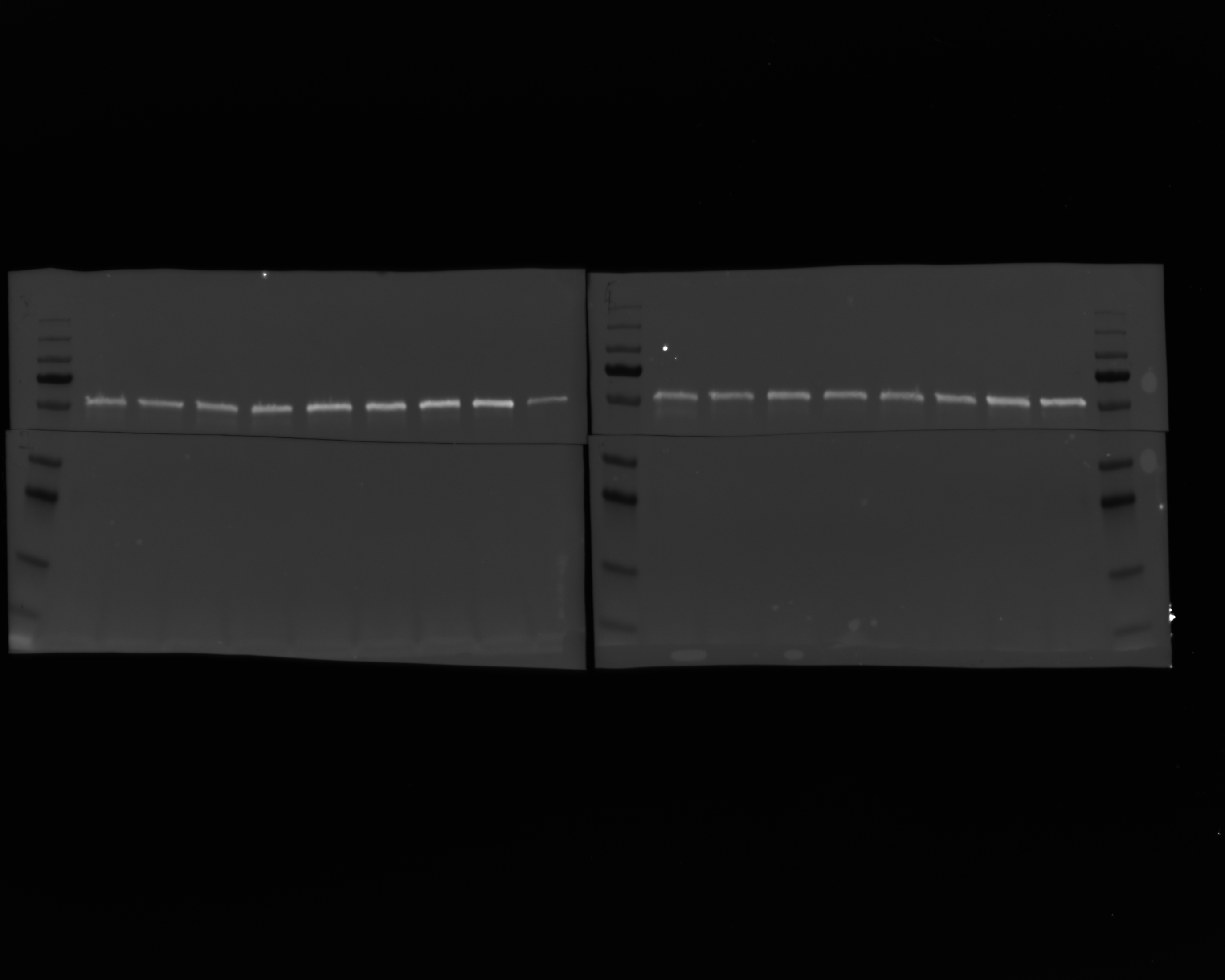

Supplement: Figure 3—source data 1. [file elife-92621-fig3-data1.zip › Figure 3- source data 1 - raw data 16bits tifs/prote-n2bleo-ir-mb2-5gh2ax-xrcc6 2021-10-14 15h24m25s Tubulin (StarBright B520).tif]

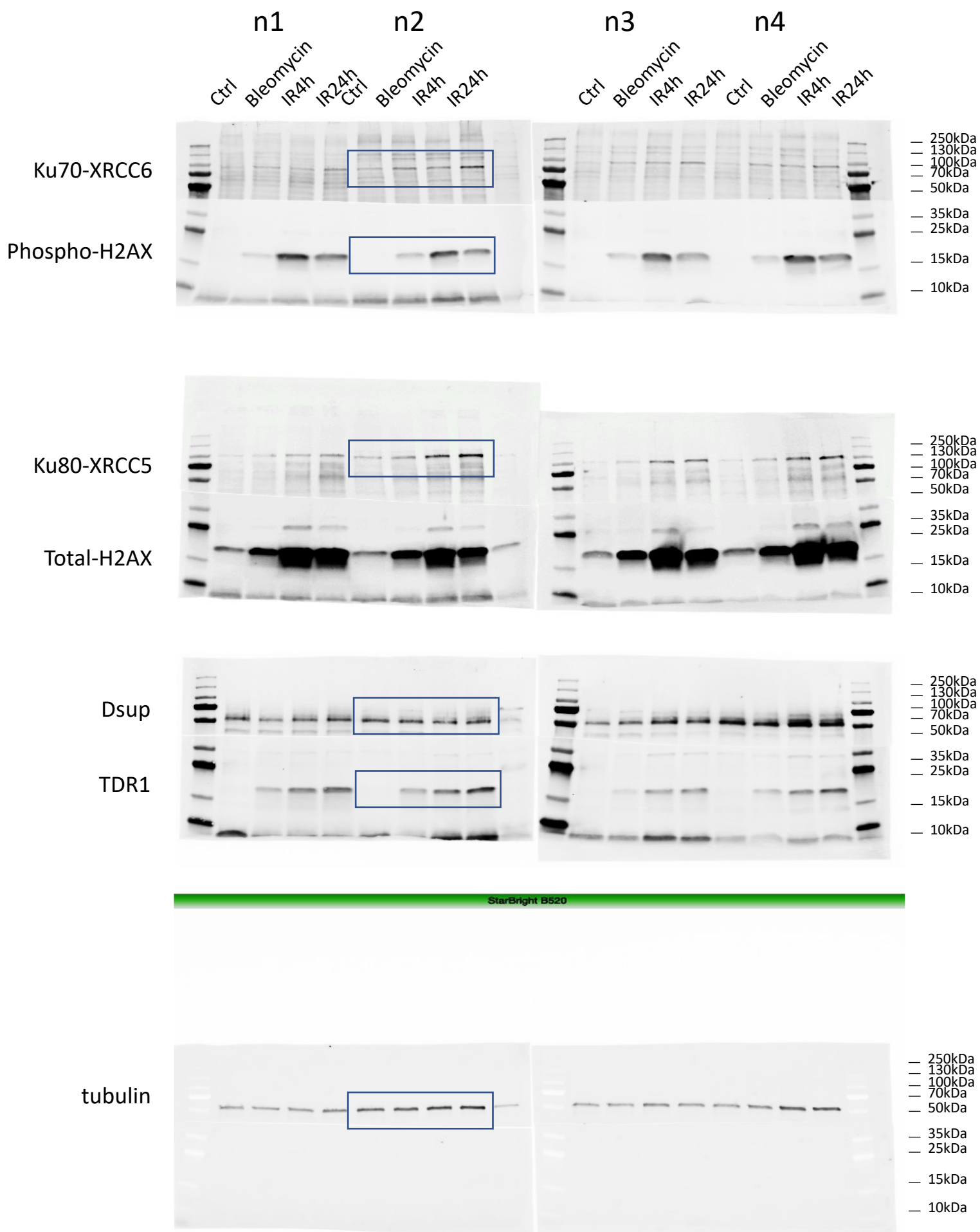

Supplement: Figure 3—source data 2. [file elife-92621-fig3-data2.pdf]

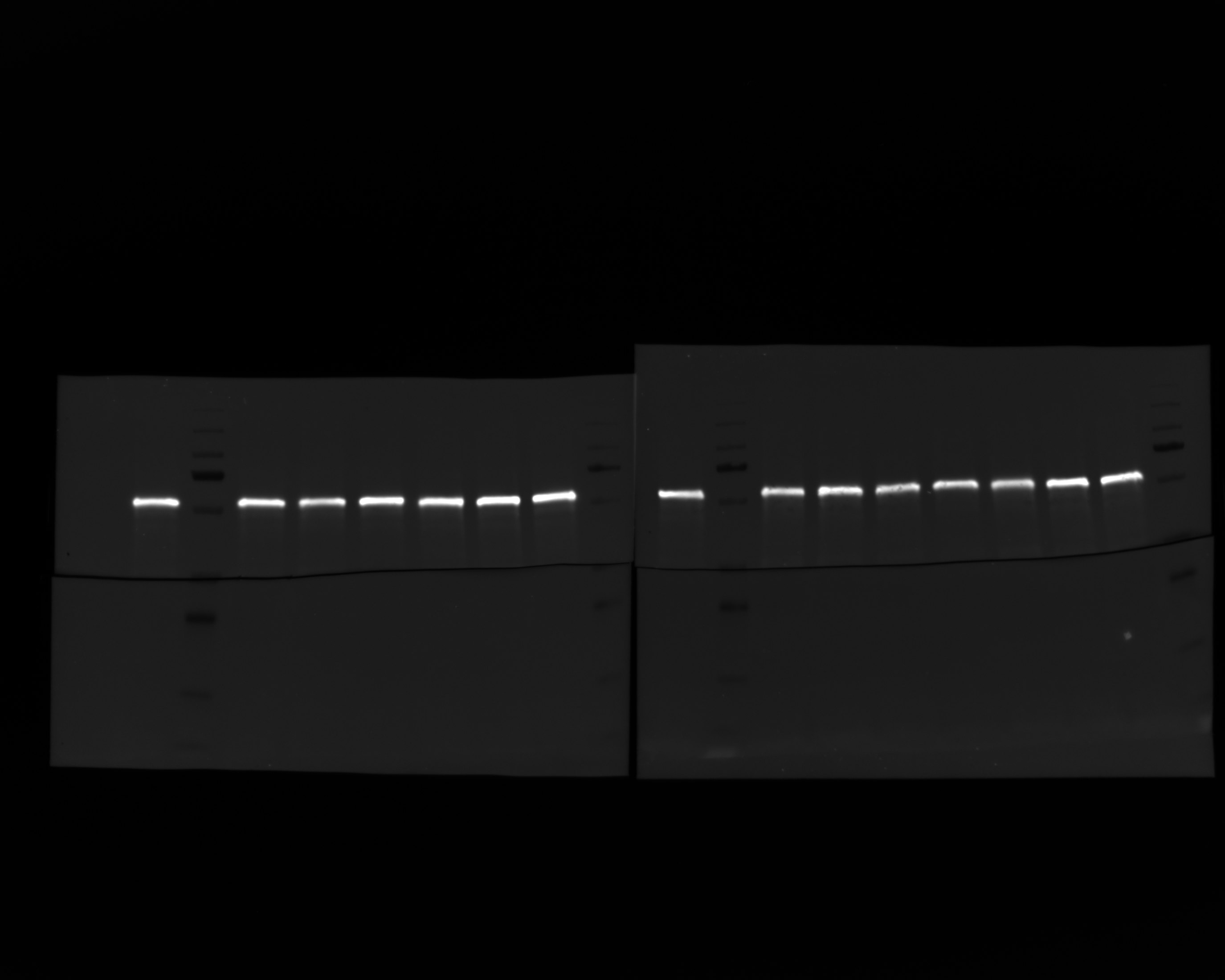

Supplement: Figure 3—figure supplement 1—source data 1. [file elife-92621-fig3-figsupp1-data1.zip › Figure 3- Figure supplement 1- source data 1 - raw data 16bits tifs/cin100-1000gy mb5-6 TDR1-Dsup 2021-09-15 15h57m57s Tubulin (StarBright B520).tif]

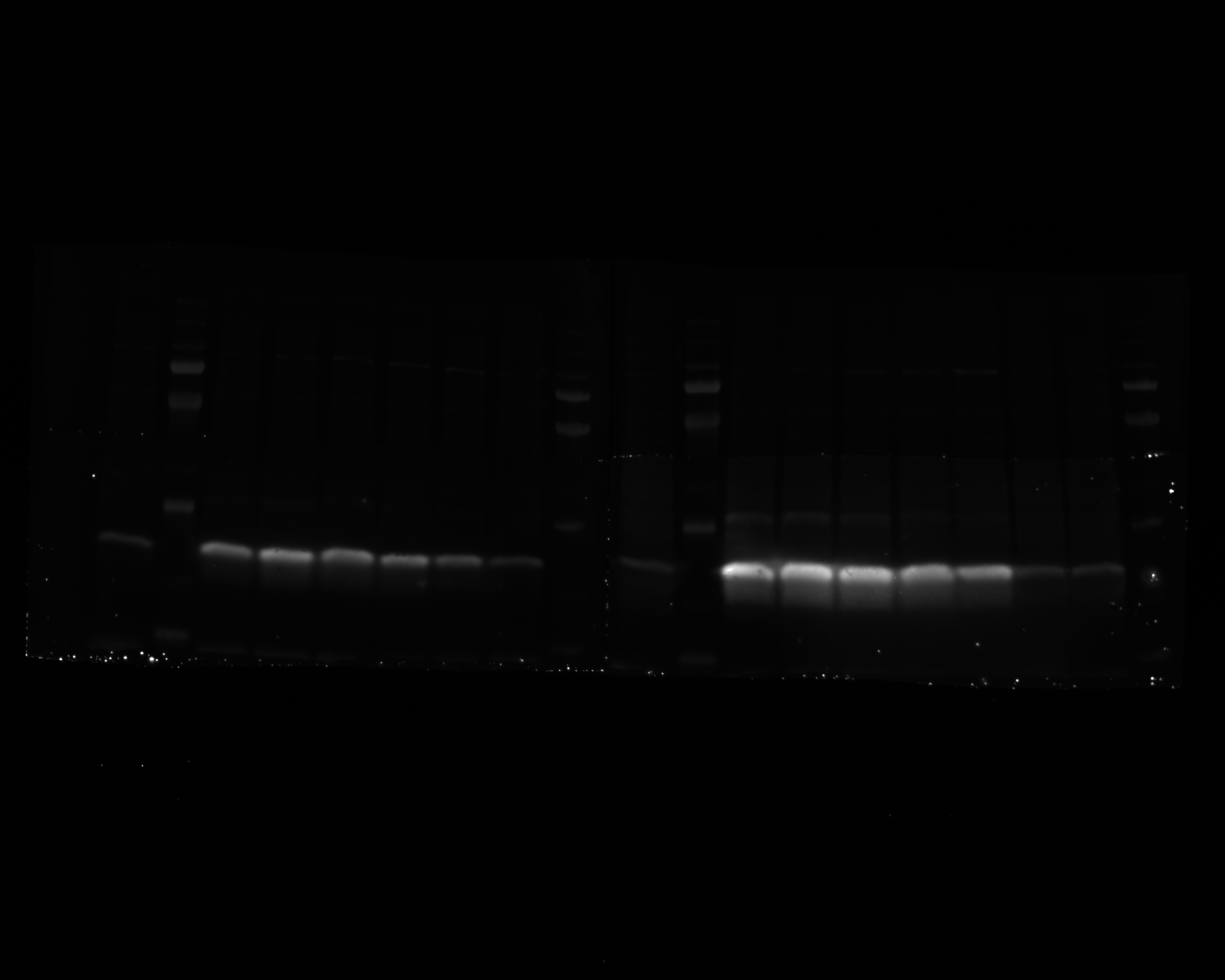

Supplement: Figure 3—figure supplement 1—source data 1. [file elife-92621-fig3-figsupp1-data1.zip › Figure 3- Figure supplement 1- source data 1 - raw data 16bits tifs/cin100-1000gy mb2-4 h2ax-xrcc6 2021-09-15 15h50m30s up Xrcc6 down H2AXtotal(StarBright B700).tif]

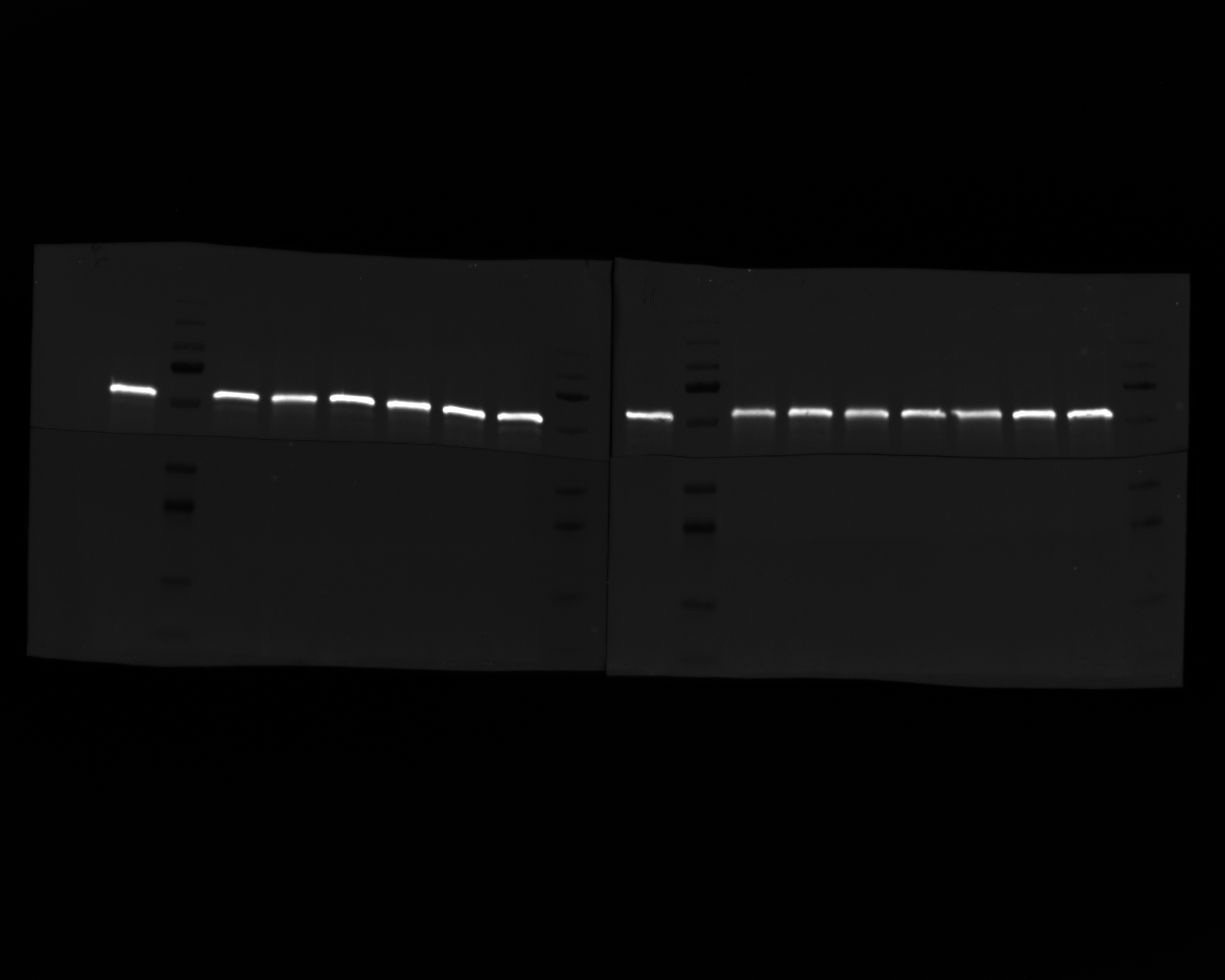

Supplement: Figure 3—figure supplement 1—source data 1. [file elife-92621-fig3-figsupp1-data1.zip › Figure 3- Figure supplement 1- source data 1 - raw data 16bits tifs/cin100-1000gy mb2-4 h2ax-xrcc6 2021-09-15 15h50m30s Tubulin (StarBright B520).tif]

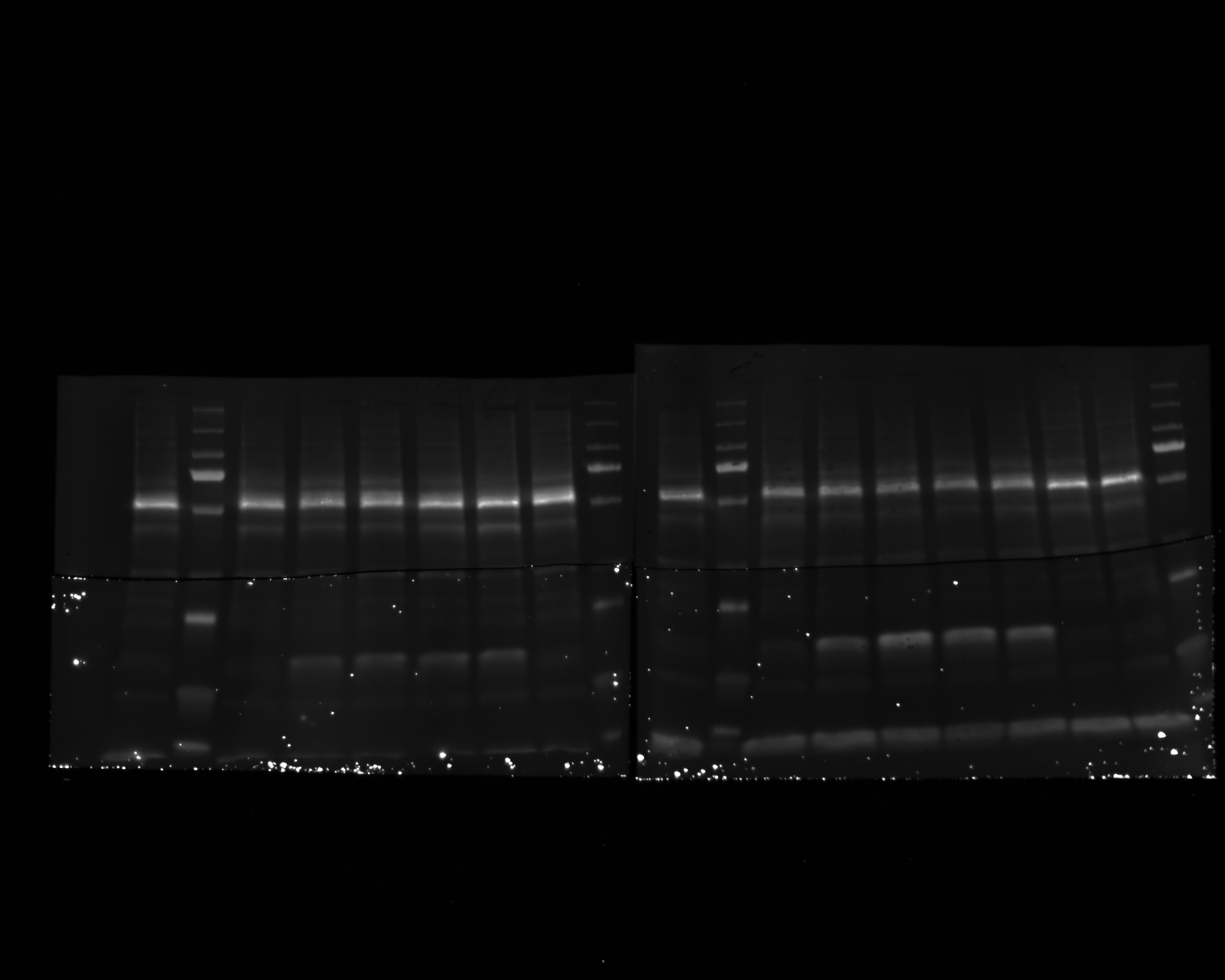

Supplement: Figure 3—figure supplement 1—source data 1. [file elife-92621-fig3-figsupp1-data1.zip › Figure 3- Figure supplement 1- source data 1 - raw data 16bits tifs/cin100-1000gy mb5-6 TDR1-Dsup 2021-09-15 15h57m57s up Dsup down TDR1(StarBright B700).tif]

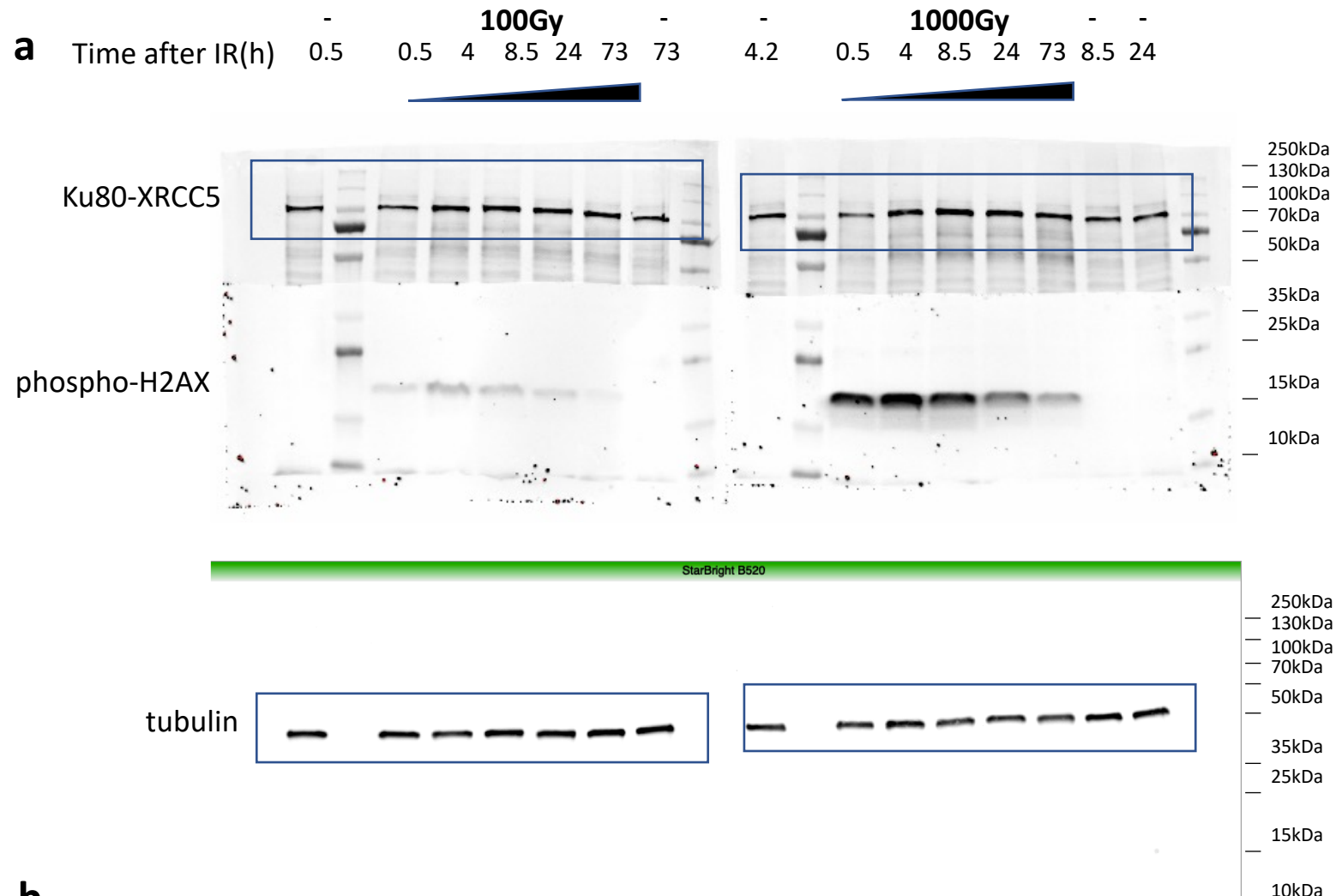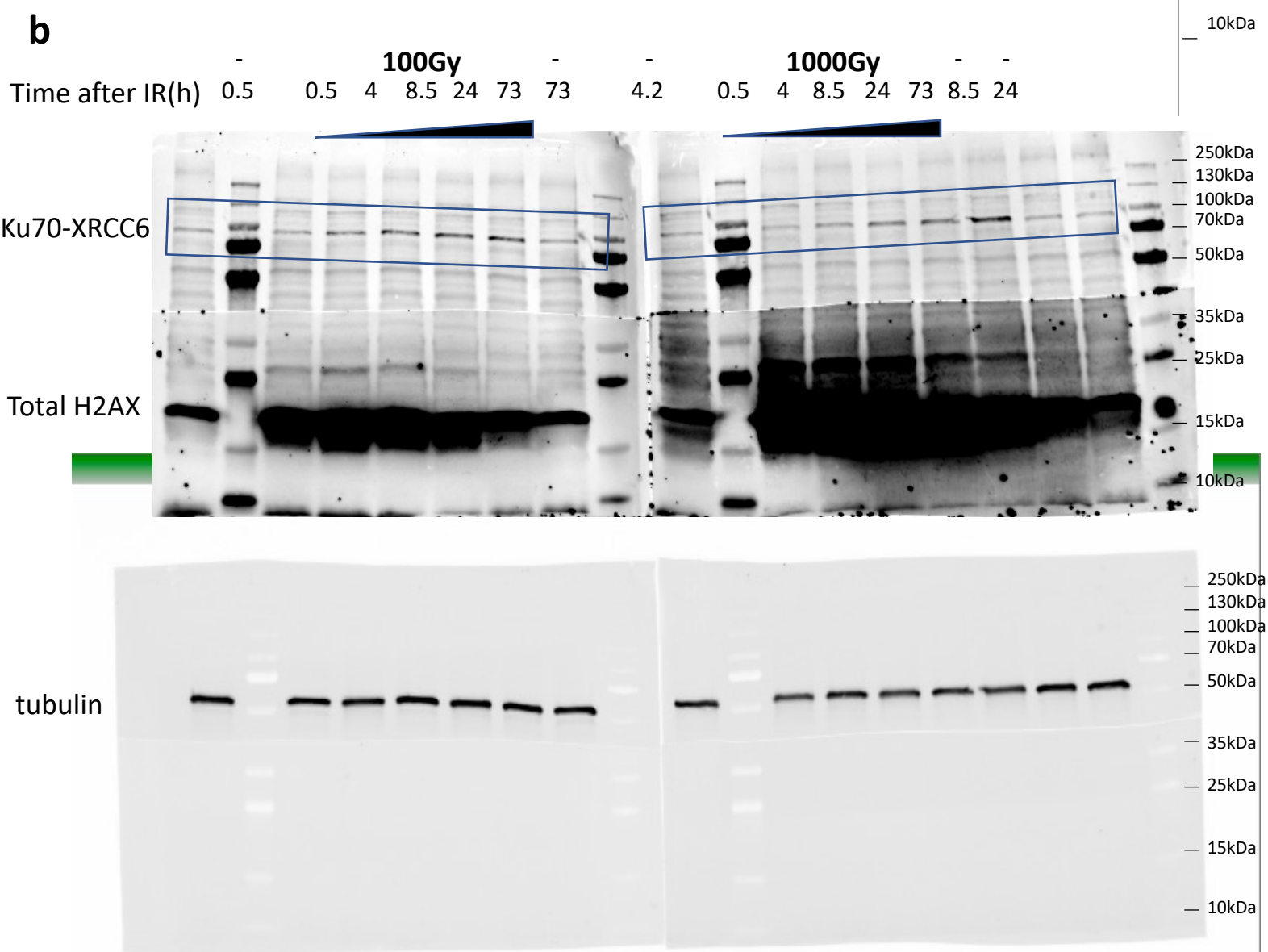

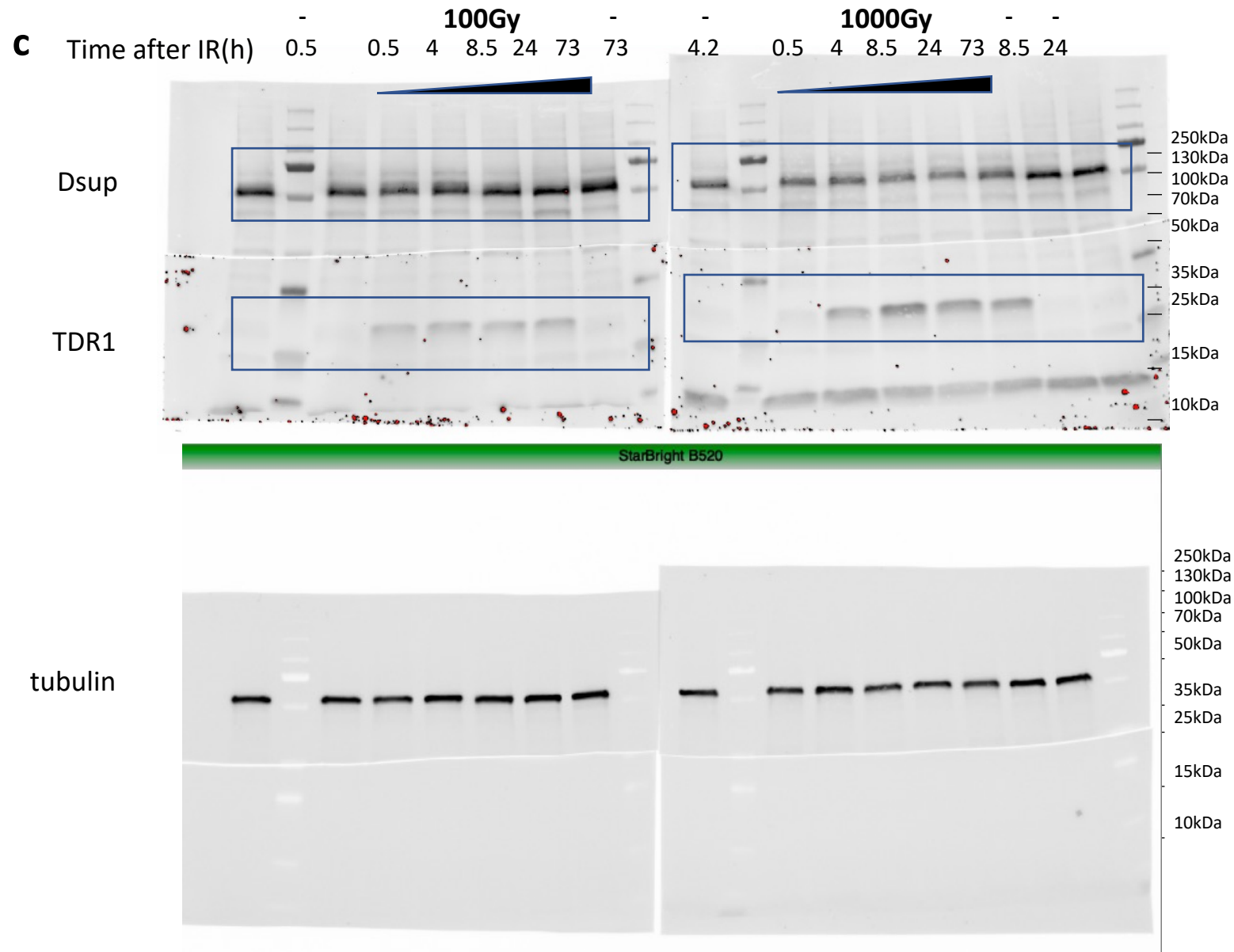

Supplement: Figure 3—figure supplement 1—source data 2. [file elife-92621-fig3-figsupp1-data2.pdf]

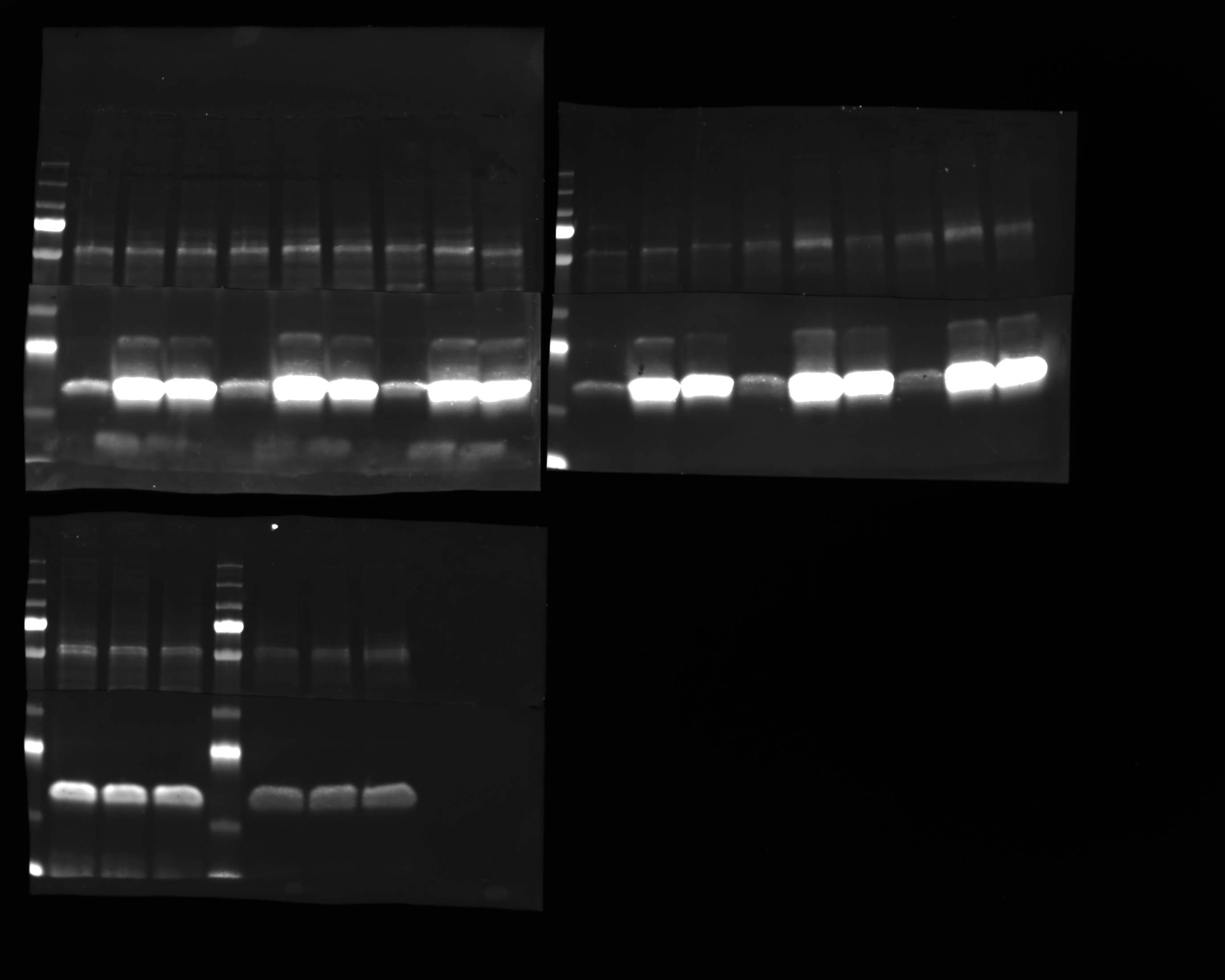

Supplement: Figure 3—figure supplement 2—source data 1. [file elife-92621-fig3-figsupp2-data1.zip › Figure 3- Figure supplement 2 - source data 1 raw data 16bits cycloheximide treatment/alpha-cyclo+-IR Dsup-H2AX ctrl4h 2022-06-30 16h44m09s up Dsup down sat H2AXtotal (StarBright B700).tif]

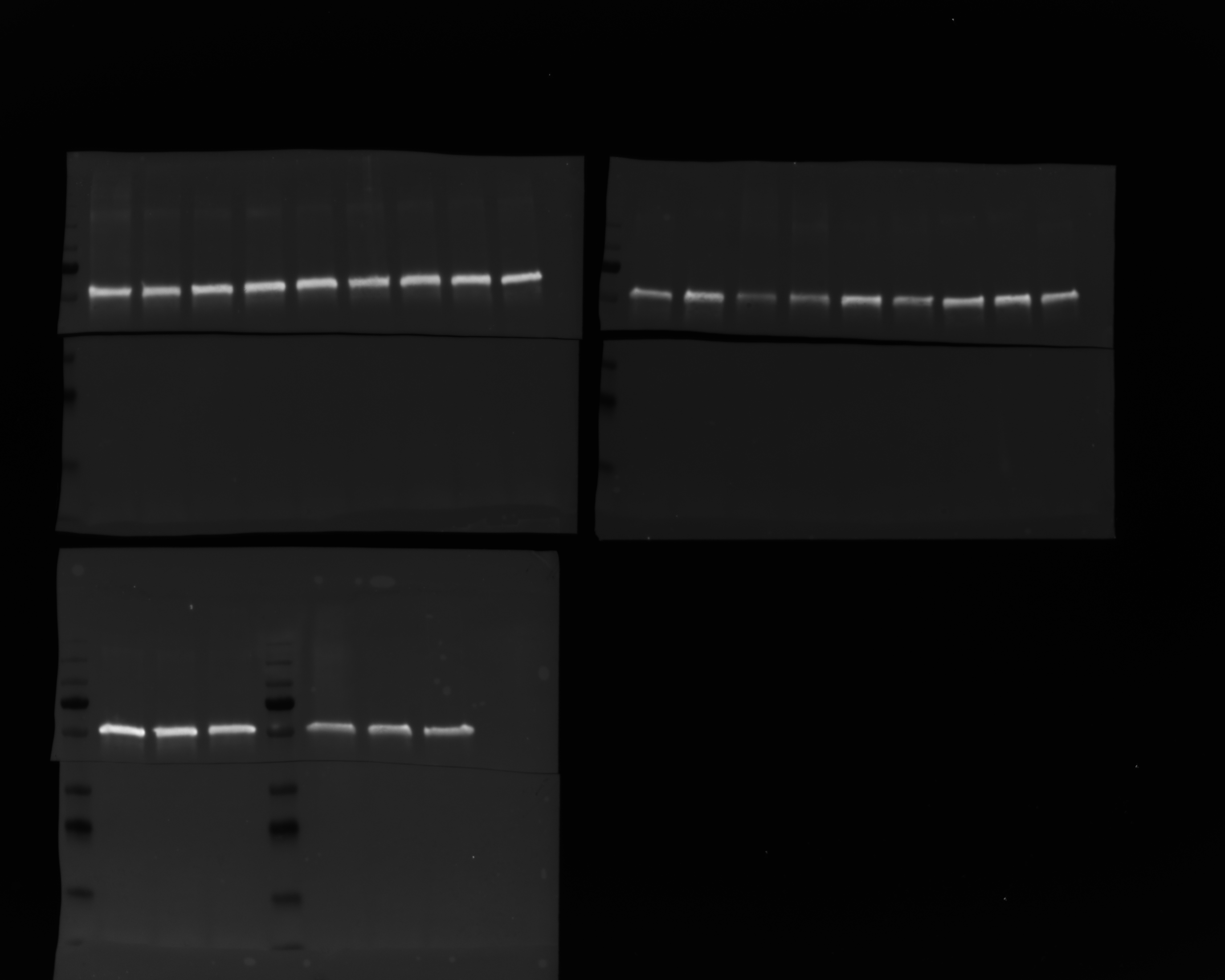

Supplement: Figure 3—figure supplement 2—source data 1. [file elife-92621-fig3-figsupp2-data1.zip › Figure 3- Figure supplement 2 - source data 1 raw data 16bits cycloheximide treatment/alpha-cyclo+-IR xrcc6-TDR1 ctrl4h 2022-06-30 16h49m25s Tubulin (StarBright B520).tif]

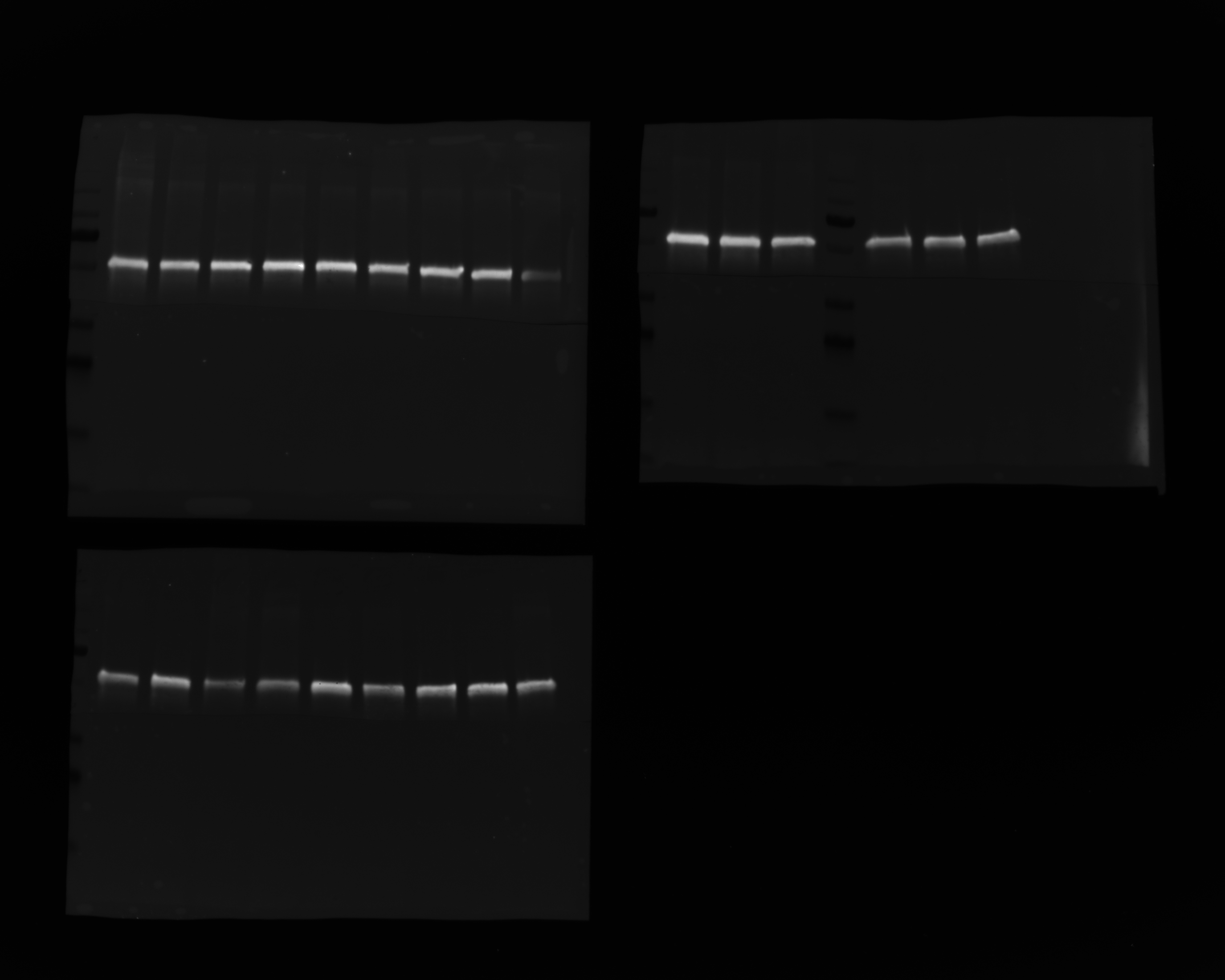

Supplement: Figure 3—figure supplement 2—source data 1. [file elife-92621-fig3-figsupp2-data1.zip › Figure 3- Figure supplement 2 - source data 1 raw data 16bits cycloheximide treatment/alpha-cyclo+-IR xrcc5-gH2AX ctrl4h 2022-07-01 09h55m01s Tubulin (StarBright B520).tif]

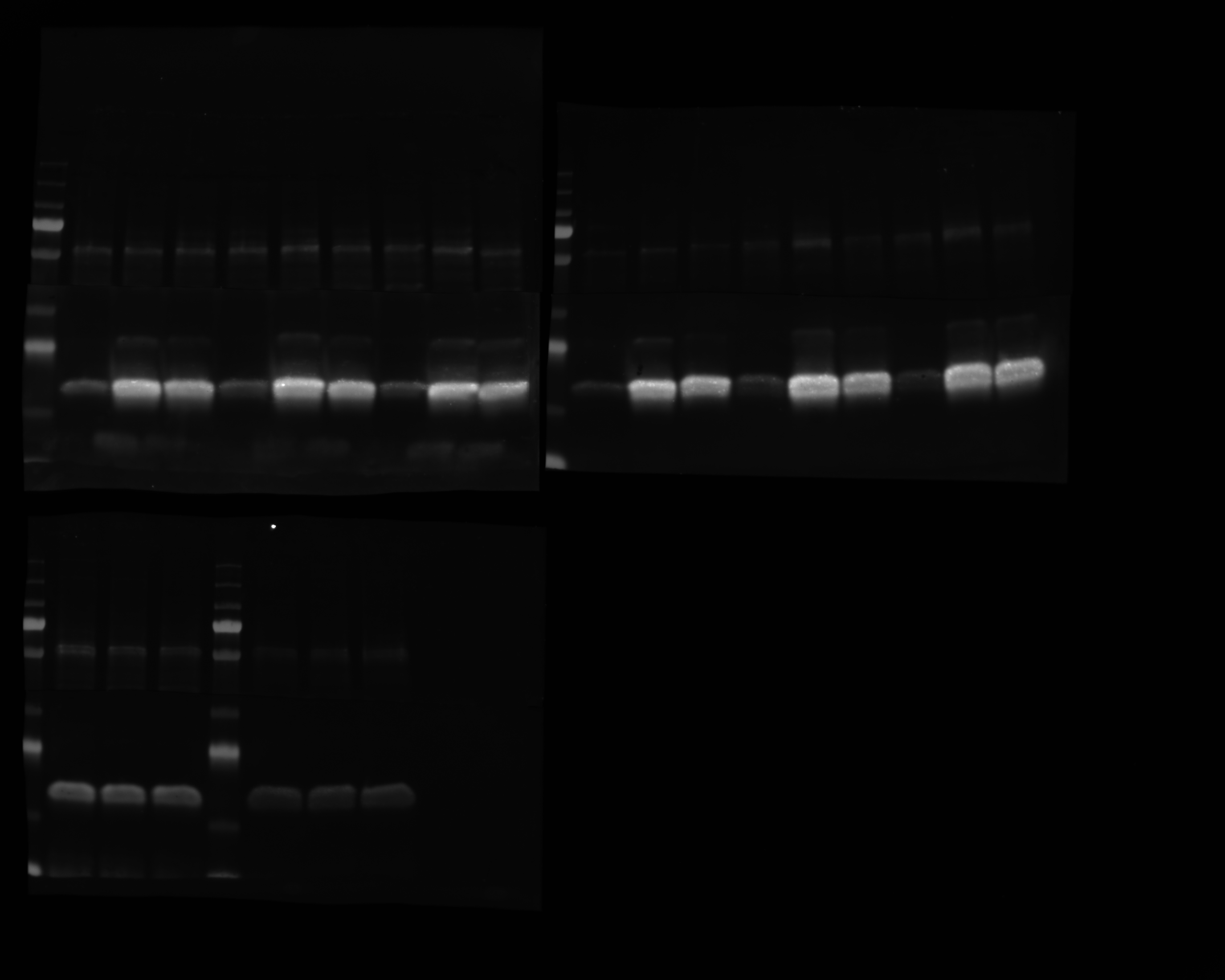

Supplement: Figure 3—figure supplement 2—source data 1. [file elife-92621-fig3-figsupp2-data1.zip › Figure 3- Figure supplement 2 - source data 1 raw data 16bits cycloheximide treatment/alpha-cyclo+-IR xrcc5-gH2AX ctrl4h 2022-06-30 16h44m55s up Xrcc5 down phosphoH2AX (StarBright B700).tif]

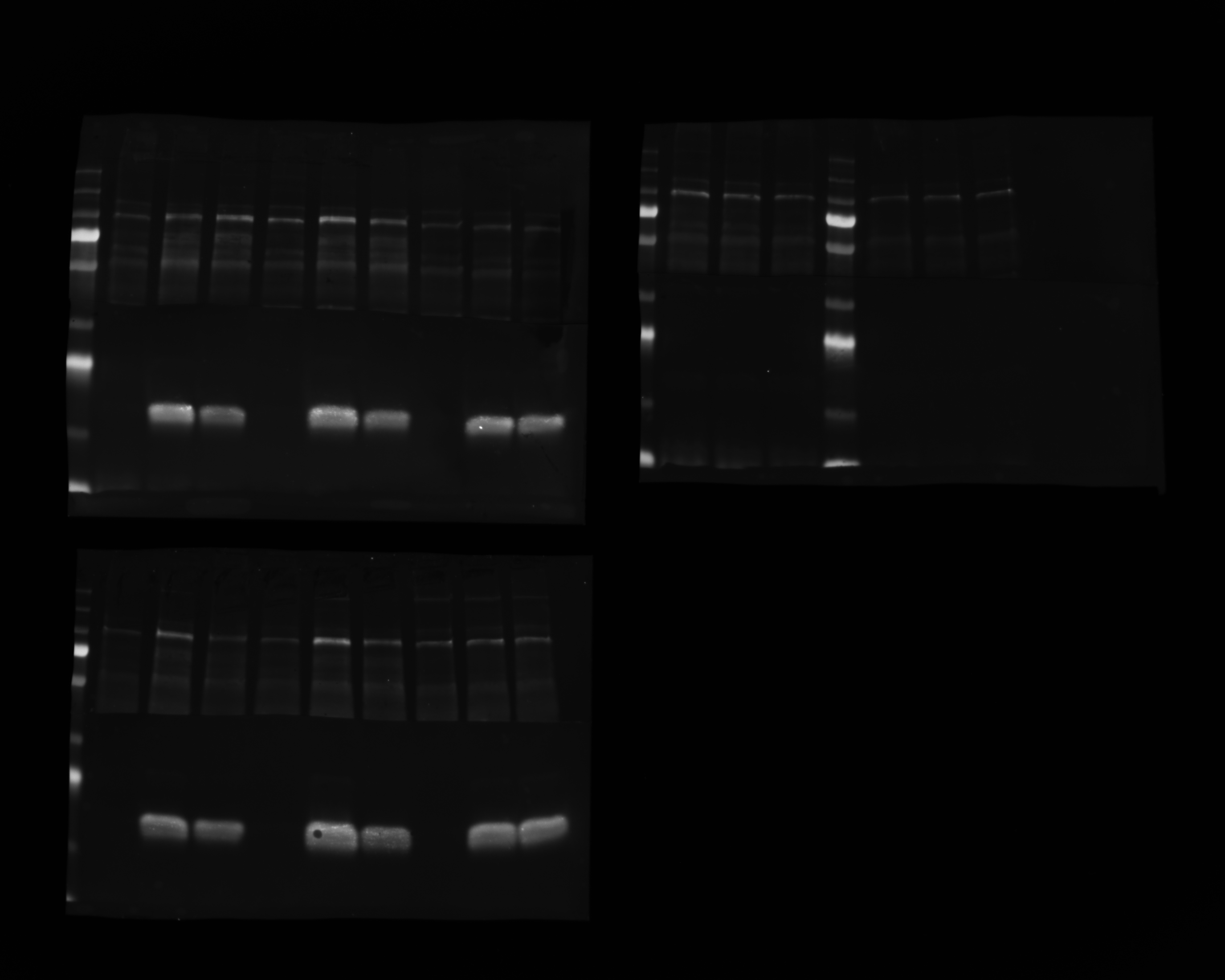

Supplement: Figure 3—figure supplement 2—source data 1. [file elife-92621-fig3-figsupp2-data1.zip › Figure 3- Figure supplement 2 - source data 1 raw data 16bits cycloheximide treatment/alpha-cyclo+-IR xrcc5-gH2AX ctrl4h 2022-07-01 09h55m01s up Xrcc5 down sat phosphoH2AX (StarBright B700).tif]

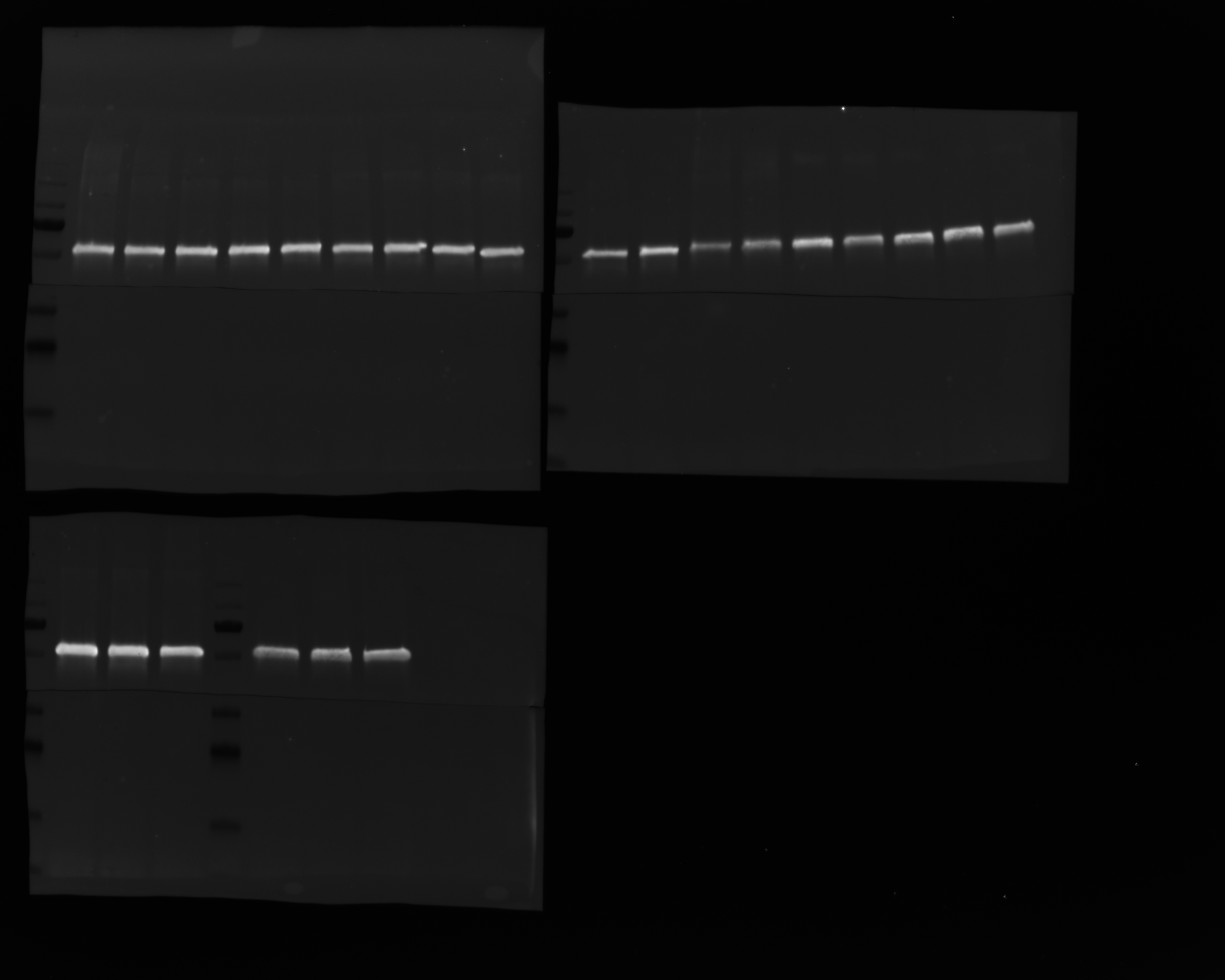

Supplement: Figure 3—figure supplement 2—source data 1. [file elife-92621-fig3-figsupp2-data1.zip › Figure 3- Figure supplement 2 - source data 1 raw data 16bits cycloheximide treatment/alpha-cyclo+-IR xrcc5-gH2AX ctrl4h 2022-06-30 16h44m55s Tubulin (StarBright B520).tif]

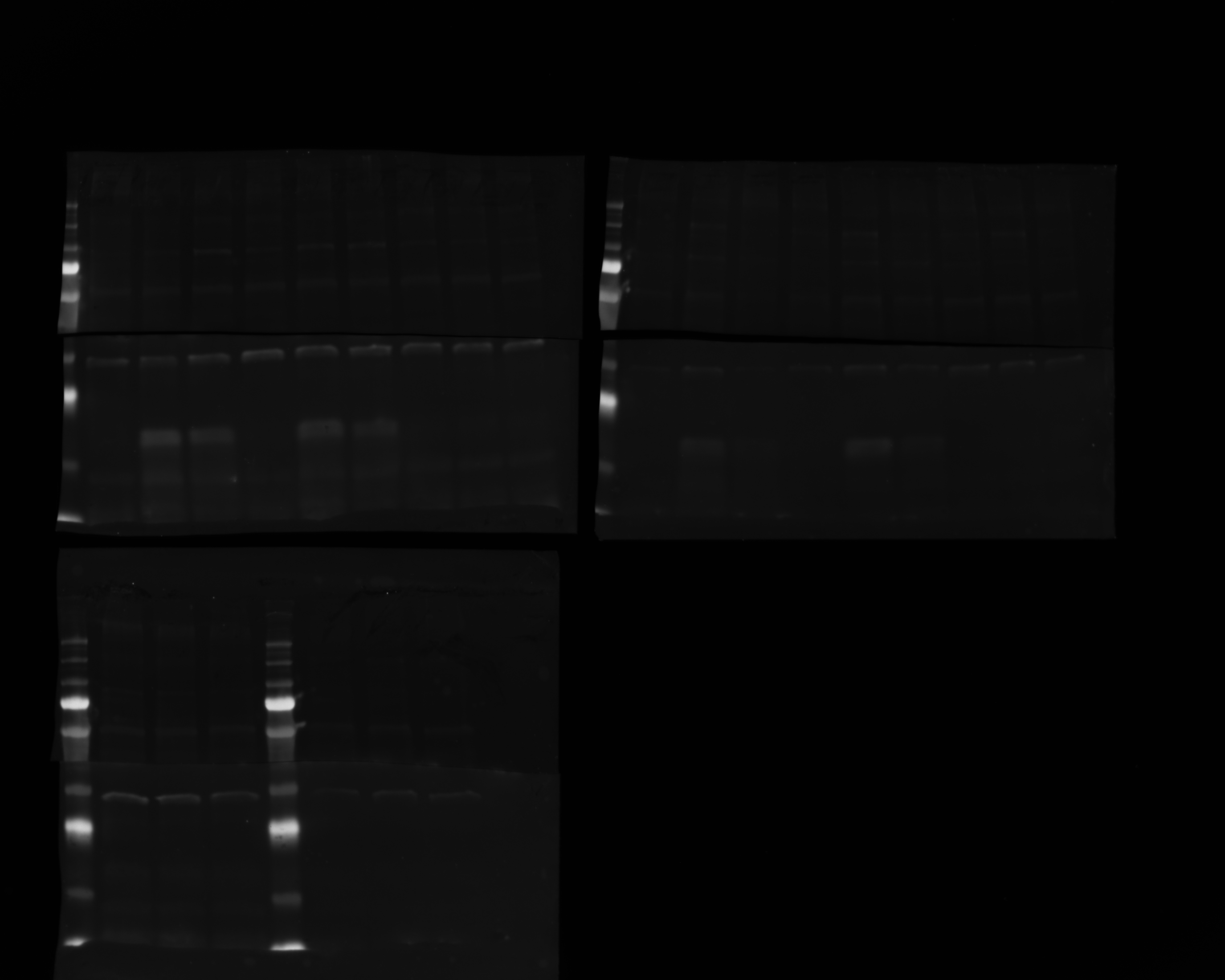

Supplement: Figure 3—figure supplement 2—source data 1. [file elife-92621-fig3-figsupp2-data1.zip › Figure 3- Figure supplement 2 - source data 1 raw data 16bits cycloheximide treatment/alpha-cyclo+-IR xrcc6-TDR1 ctrl4h 2022-06-30 16h49m25s up xrcc6 down TDR1 (StarBright B700).tif]

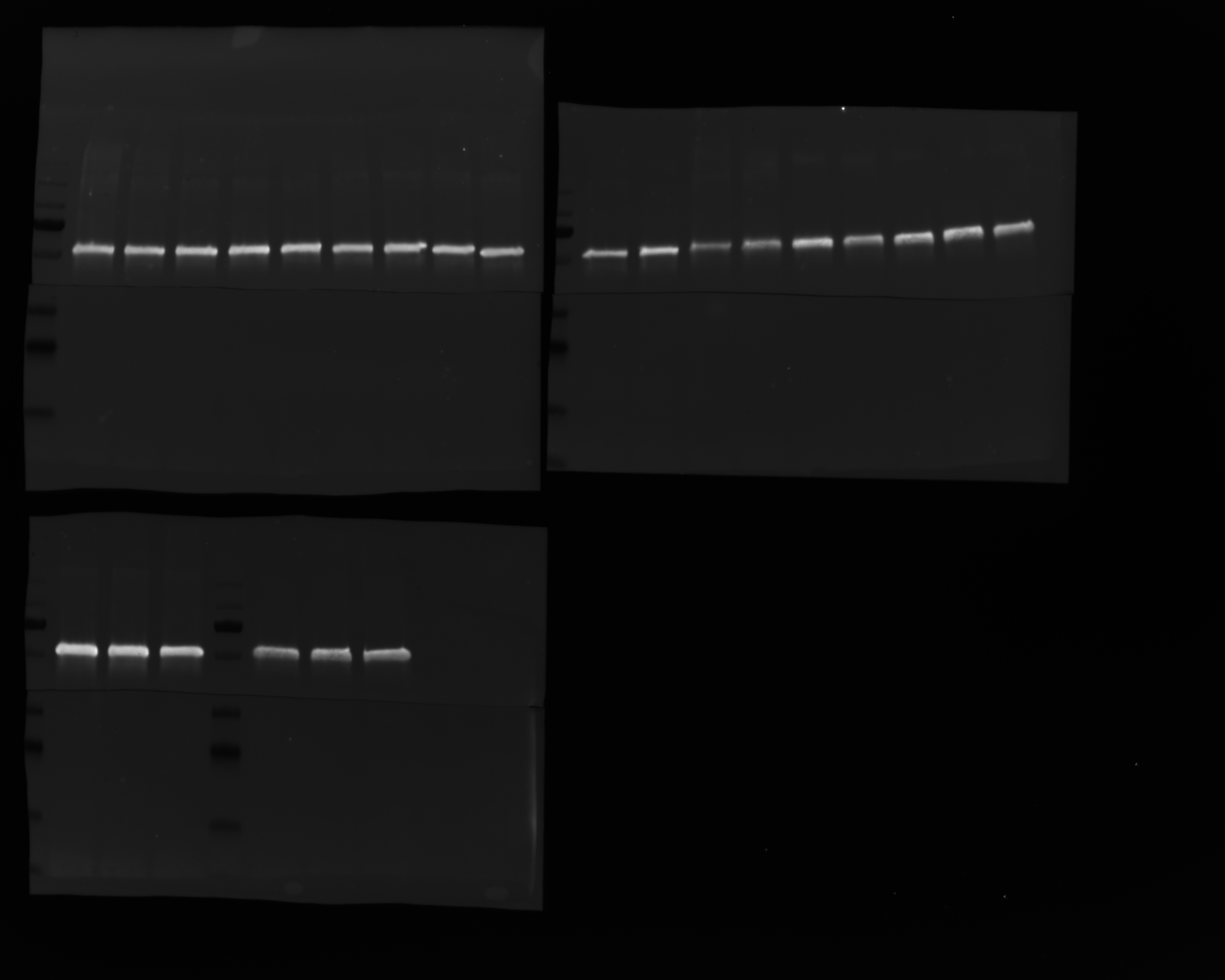

Supplement: Figure 3—figure supplement 2—source data 1. [file elife-92621-fig3-figsupp2-data1.zip › Figure 3- Figure supplement 2 - source data 1 raw data 16bits cycloheximide treatment/alpha-cyclo+-IR Dsup-H2AX ctrl4h 2022-06-30 16h44m09s Tubulin (StarBright B520).tif]

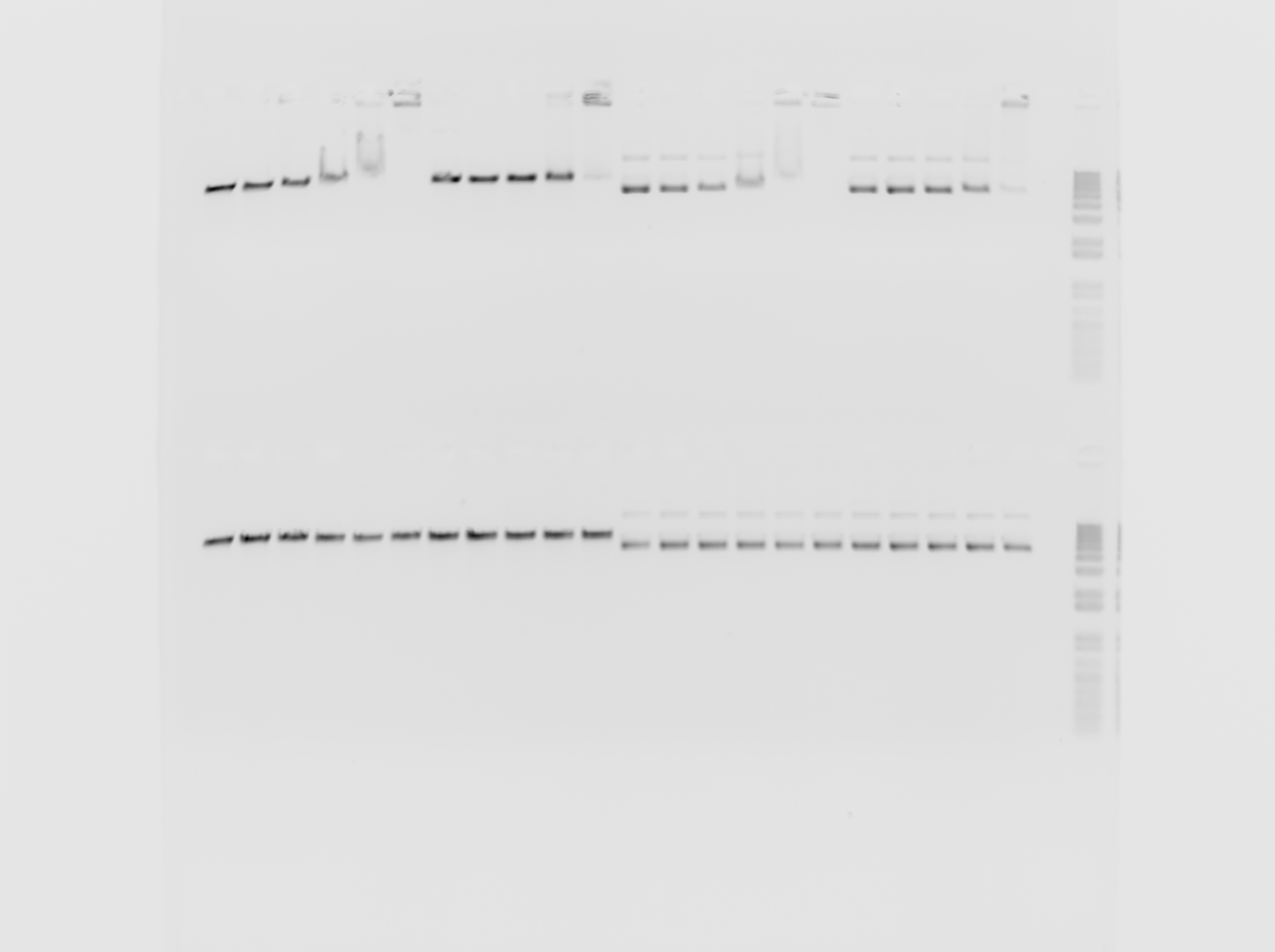

Supplement: Figure 5—source data 1. [file elife-92621-fig5-data1.zip › Figure 5- source data 1- raw data 16bit tifs.tif]

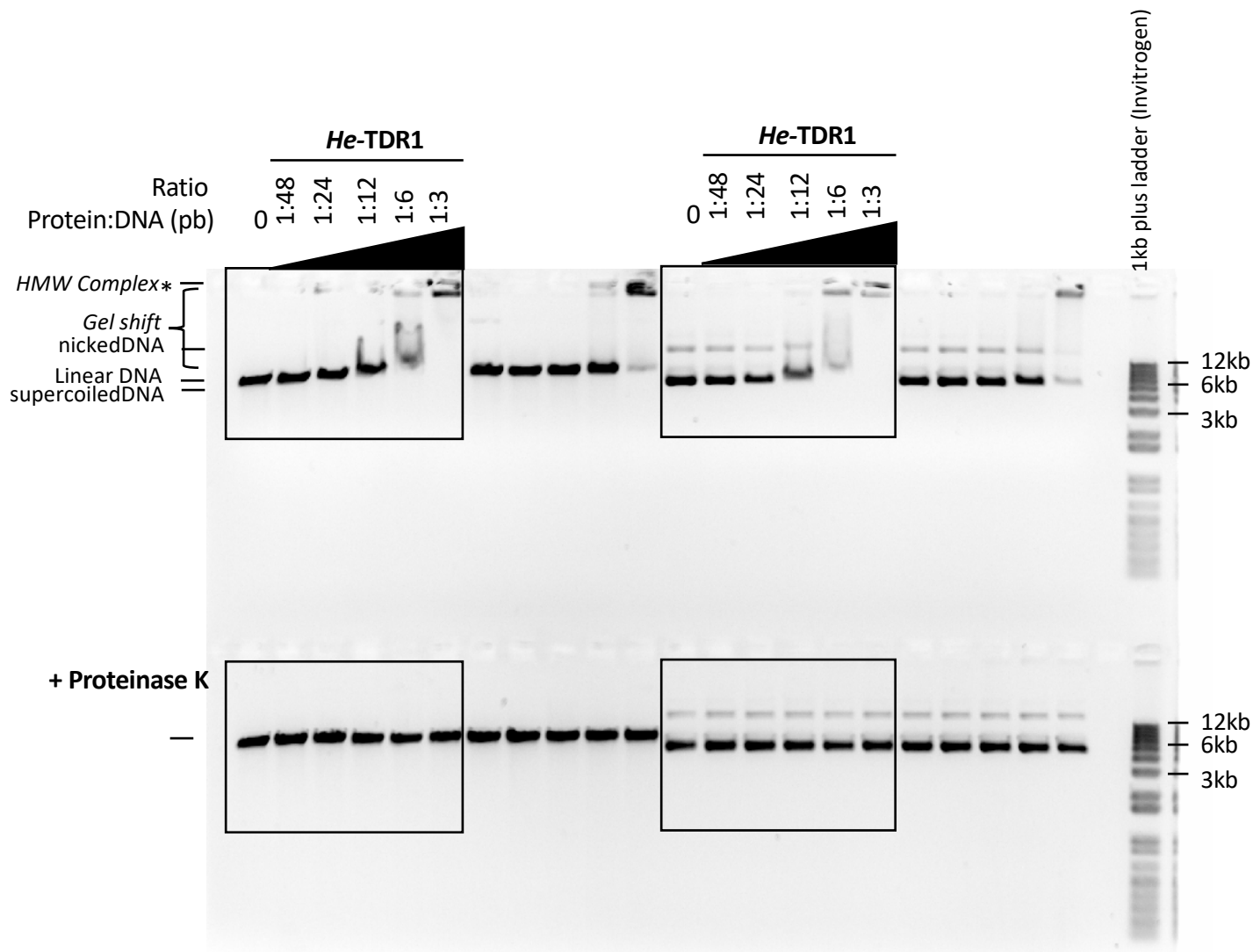

Supplement: Figure 5—source data 2. [file elife-92621-fig5-data2.pdf]

**a**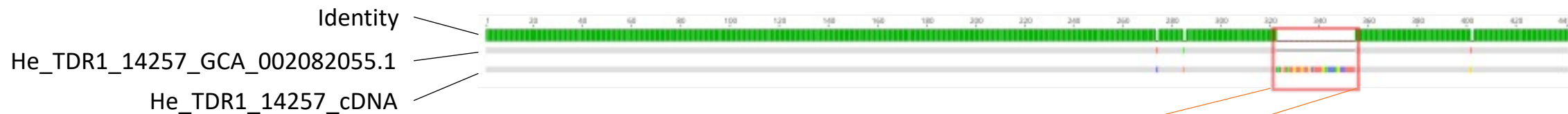**b**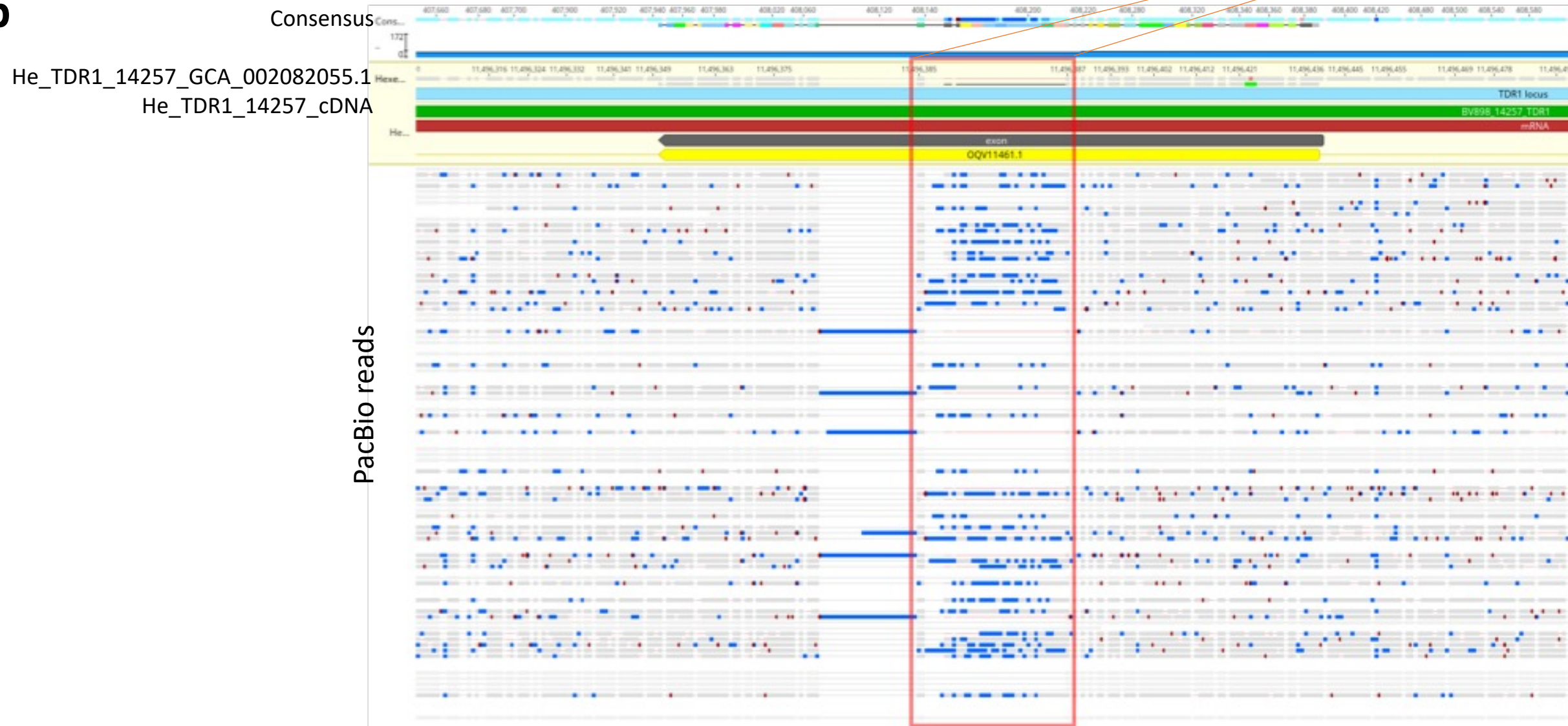

Supplement: Supplementary file 1. — (a) Alignment of H. exemplaris genome assembly GCA_002082055.1 with cDNA sequence of He-TDR1 obtained from Oxford Nanopore Technology (ONT) long read sequencing and cDNA cloning showed that a portion of TDR1 sequence is missing in the current assembly. (b) Alignment of PacBio reads used for genome assembly with H. exemplaris genome assembly GCA_002082055.1 and He-TDR1 cDNA. A zoom on the missing sequence (boxed in orange) shows the poor quality of PacBio reads used for genome assembly at this locus, likely explaining the absence of the missing He-TDR1 cDNA sequence in the current genome assembly. PacBio reads (SRX2495681, Yoshida et al., 2017) were downloaded from NCBI, mapped with minimap2 (Li, 2018) and alignment visualization was performed with Geneious Prime (v2023.1). Blue and red dots respectively indicate mismatches and indels in the alignment. cDNA sequence of He-TDR1 is provided in Supplementary file 2 and encodes for a 146 amino acid long protein. [file elife-92621-supp1.pdf]

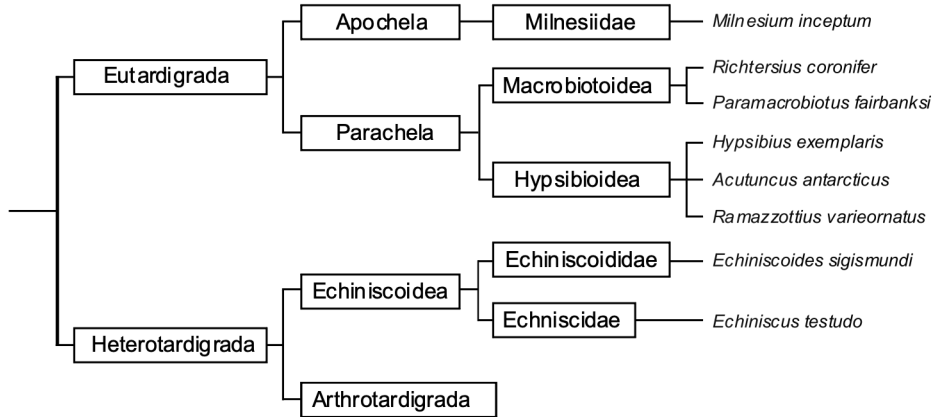

| Nucleus                                                                             |                                                                                     | Other cellular compartments                                                         |                                                                                     |                                                                                     |                                                                                     |                                                                                     | Secreted                                                                            |
|-------------------------------------------------------------------------------------|-------------------------------------------------------------------------------------|-------------------------------------------------------------------------------------|-------------------------------------------------------------------------------------|-------------------------------------------------------------------------------------|-------------------------------------------------------------------------------------|-------------------------------------------------------------------------------------|-------------------------------------------------------------------------------------|
| TDR1                                                                                | Dsup                                                                                | AMNP                                                                                | MAHS                                                                                | CAHS                                                                                | EtAHS-A                                                                             | EtAHS-B                                                                             | SAHS                                                                                |
| 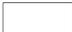 | 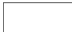 | 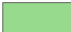 | 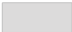 | 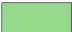 | 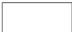 | 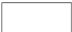 | 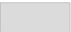 |
| 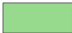 | 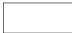 | 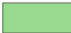 | 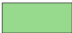 | 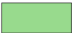 | 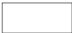 | 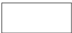 | 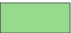 |
| 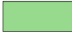 | 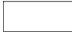 | 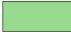 | 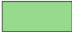 | 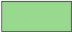 | 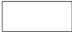 | 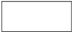 | 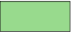 |
| 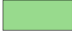 | 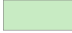 | 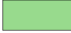 | 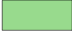 | 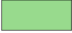 | 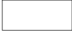 | 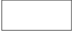 | 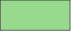 |
| 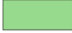 | 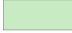 | 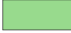 | 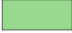 | 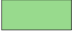 | 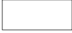 | 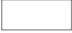 | 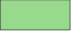 |
| 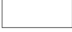 | 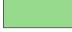 | 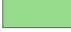 | 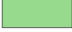 | 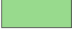 | 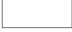 | 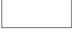 | 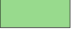 |
| 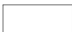 | 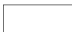 | 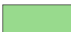 | 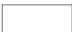 | 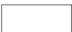 | 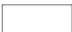 | 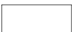 | 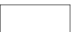 |
| 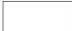 | 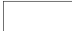 | 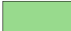 | 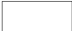 | 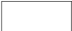 | 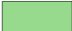 | 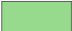 | 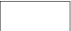 |

Supplement: Supplementary file 6. — Green and white boxes indicate presence and absence, respectively, of the indicated gene or gene family as found in Arakawa, 2022, and in this work for TDR1. Light green indicates presence of potential Rv-Dsup ortholog with hypothetical function in radio-resistance (Arakawa, 2022). The figure in Supplementary file 6 is adapted from Figure 3 of Arakawa, 2022, and augmented with additional information from this work. A TDR1 homolog could not be identified by BLAST analysis of R. varieornatus genome and available transcriptomics data. Sequence similarity of a potential TDR1 protein in R. varieornatus may be too low and indicate alternative mechanisms of radio-resistance in R. varieornatus, e.g., based on stronger activity of the Rv-Dsup compared to He- and Aa-Dsup. Investigation in additional species may help to clarify the presence/absence of TDR1 in the Ramazzottius genus. [file elife-92621-supp6.pdf]
