# Supplementary material for: Comparative transcriptomics reveal a novel tardigrade-specific DNA-binding protein induced in response to ionizing radiation
Source: eLife. 2024 Jul 9;13:RP92621. doi: 10.7554/eLife.92621 (PMC11233135; doi:10.7554/eLife.92621)
Supplement: Figure 3—figure supplement 2—source data 2. [file elife-92621-fig3-figsupp2-data2.pdf]

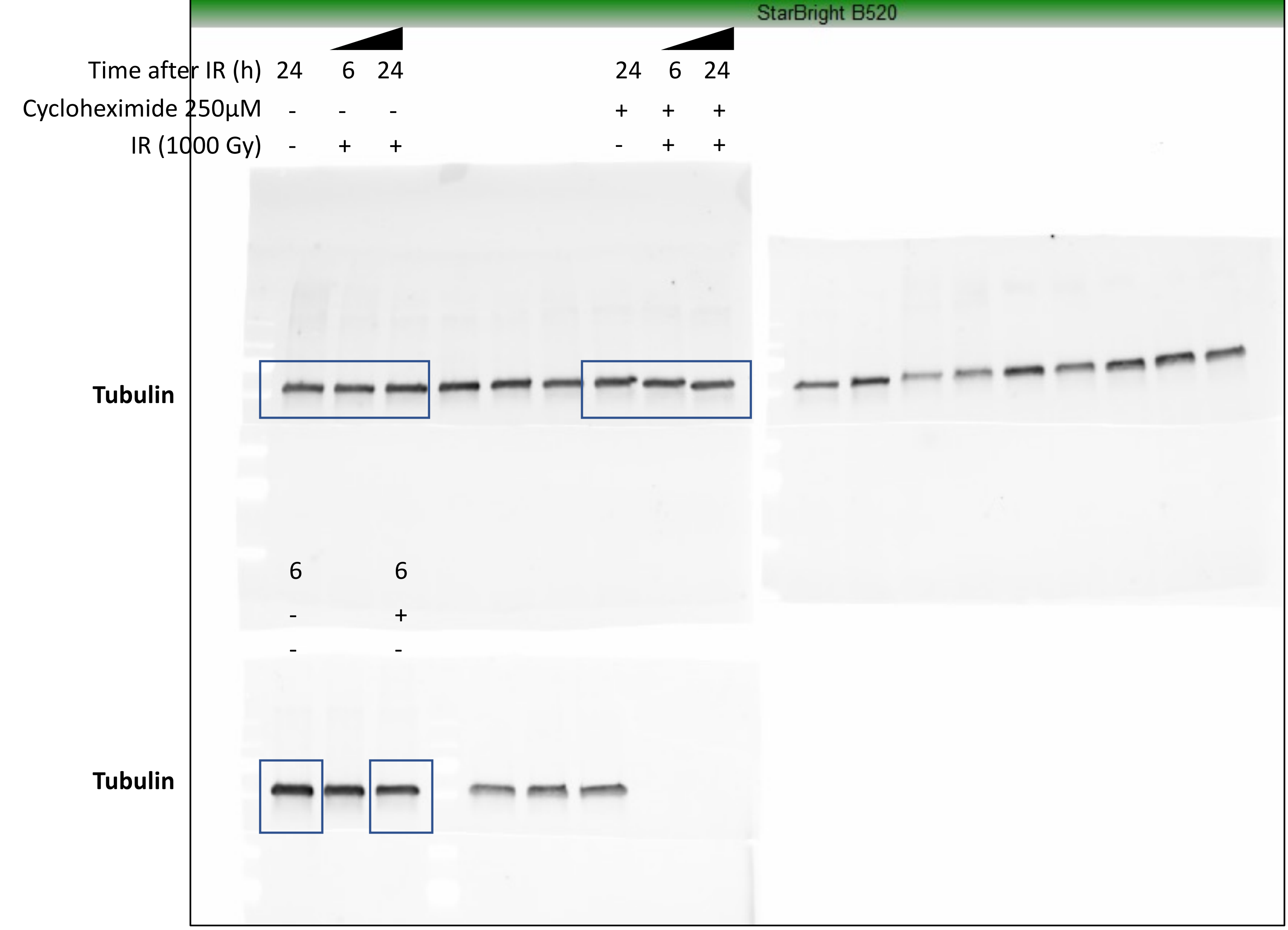
StarBright B520
Time after IR (h) 24 6 24 24 6 24
Cycloheximide 250µM - - - + + +
IR (1000 Gy) - + + - + +
Tubulin
6 6
- +
- -
Tubulin

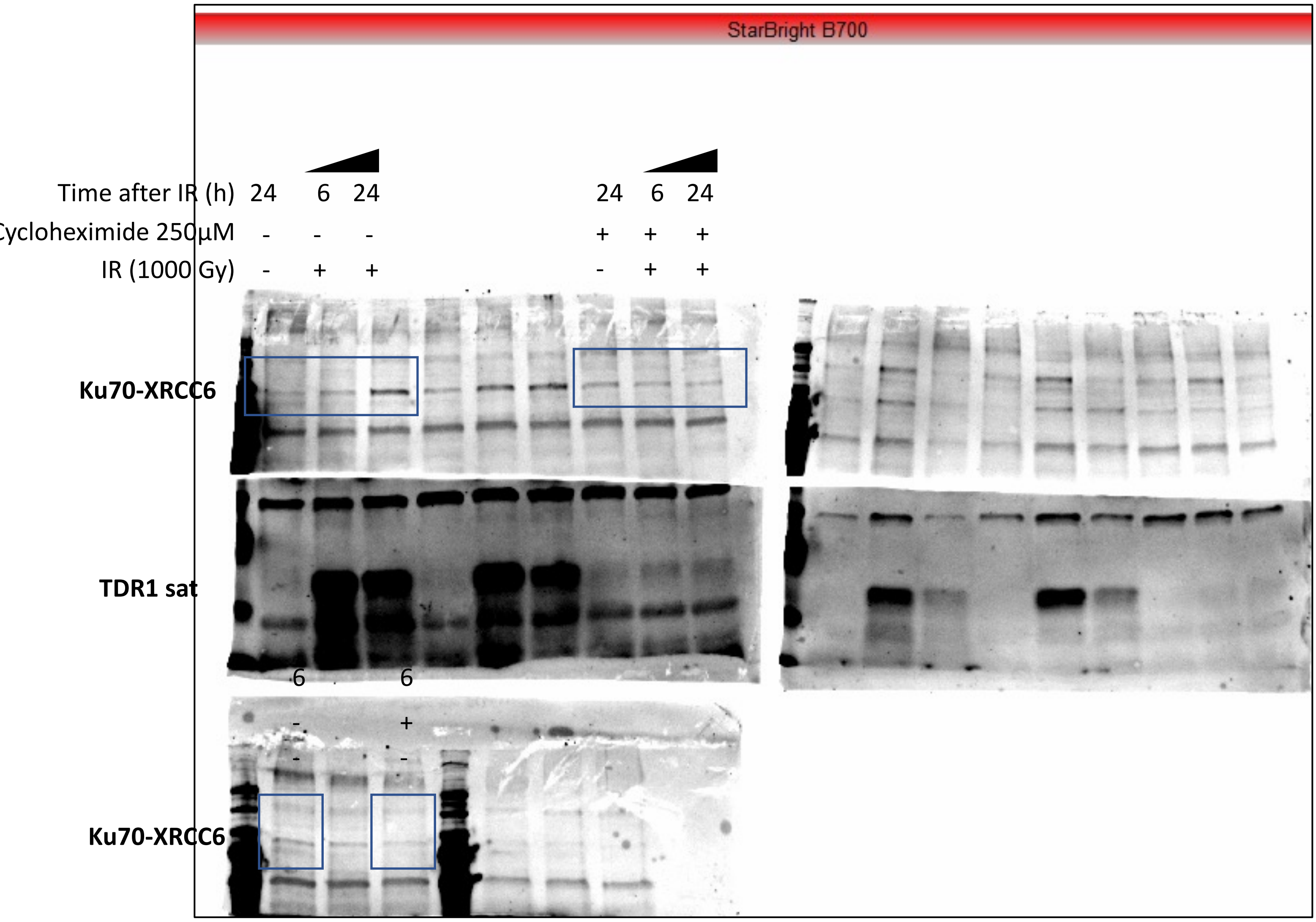
StarBright B700
Time after IR (h) 24 6 24 24 6 24
Cycloheximide 250µM - - - + + +
IR (1000 Gy) - + + - + +
Ku70-XRCC6
TDR1 sat
6 6
- +
- -
Ku70-XRCC6

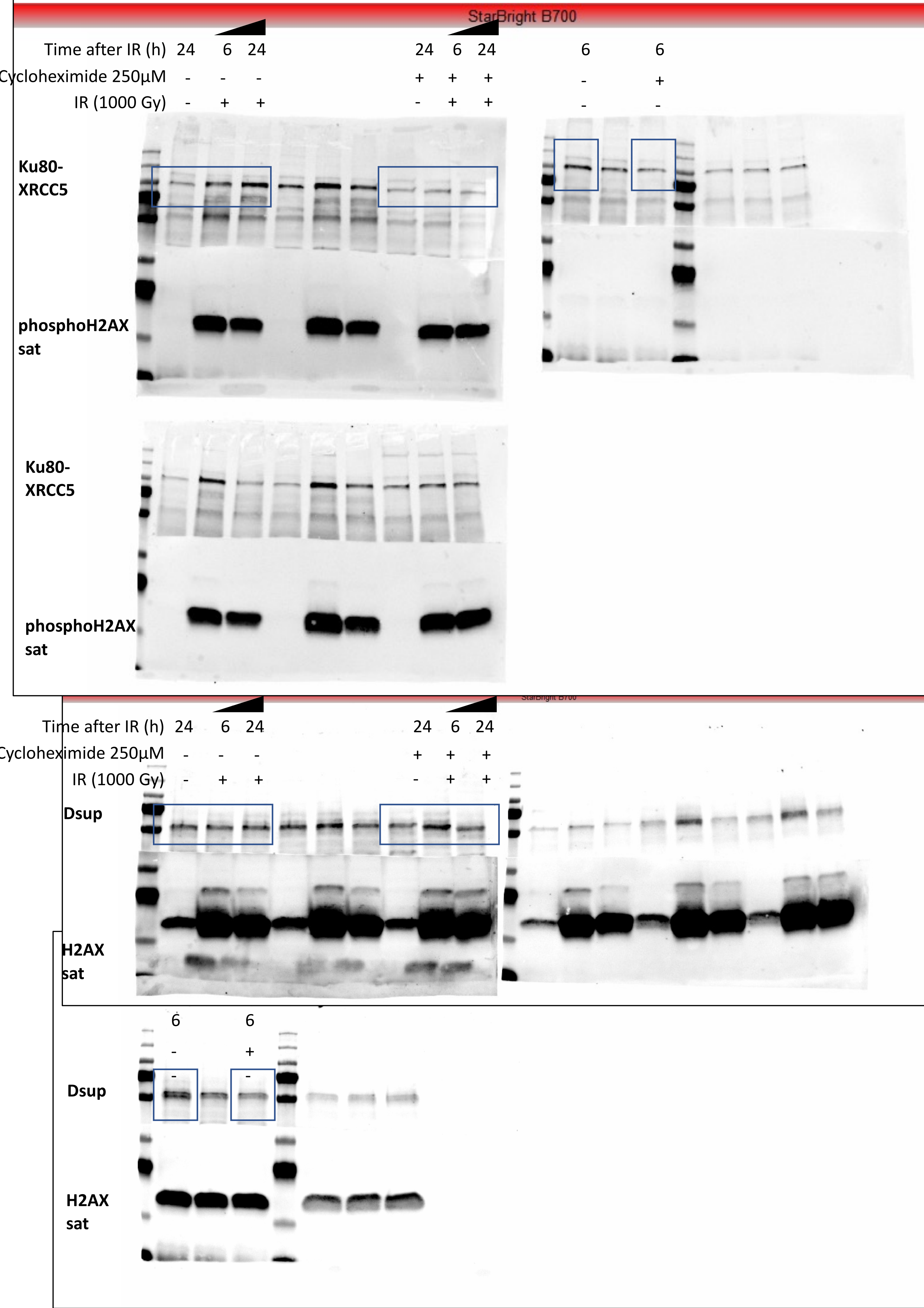
StarBright B700
Time after IR (h) 24 6 24 24 6 24 6 6
Cycloheximide 250µM - - - + + + - +
IR (1000 Gy) - + + - + + - -
Ku80-XRCC5
phosphoH2AX sat
Ku80-XRCC5
phosphoH2AX sat
Time after IR (h) 24 6 24 24 6 24
Cycloheximide 250µM - - - + + +
IR (1000 Gy) - + + - + +
Dsup
H2AX sat
6 6
- +
- -
Dsup
H2AX sat

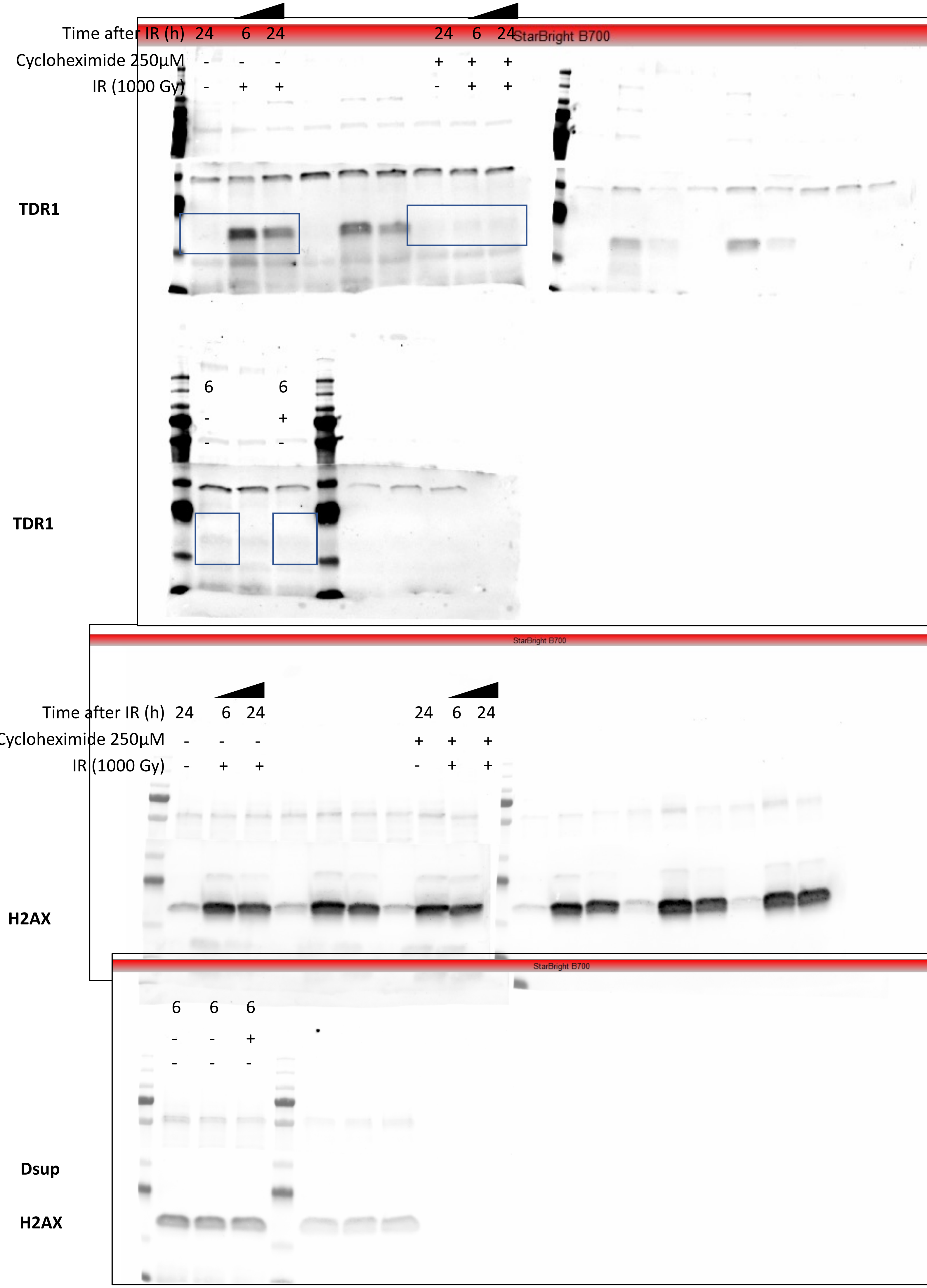
Time after IR (h) 24 6 24 24 6 24
Cycloheximide 250µM - - - + + +
IR (1000 Gy) - + + - + +
StarBright B700
TDR1
6 6
- +
- -
TDR1
StarBright B700
Time after IR (h) 24 6 24 24 6 24
Cycloheximide 250µM - - - + + +
IR (1000 Gy) - + + - + +
H2AX
StarBright B700
6 6 6
- - +
- - -
Dsup
H2AX

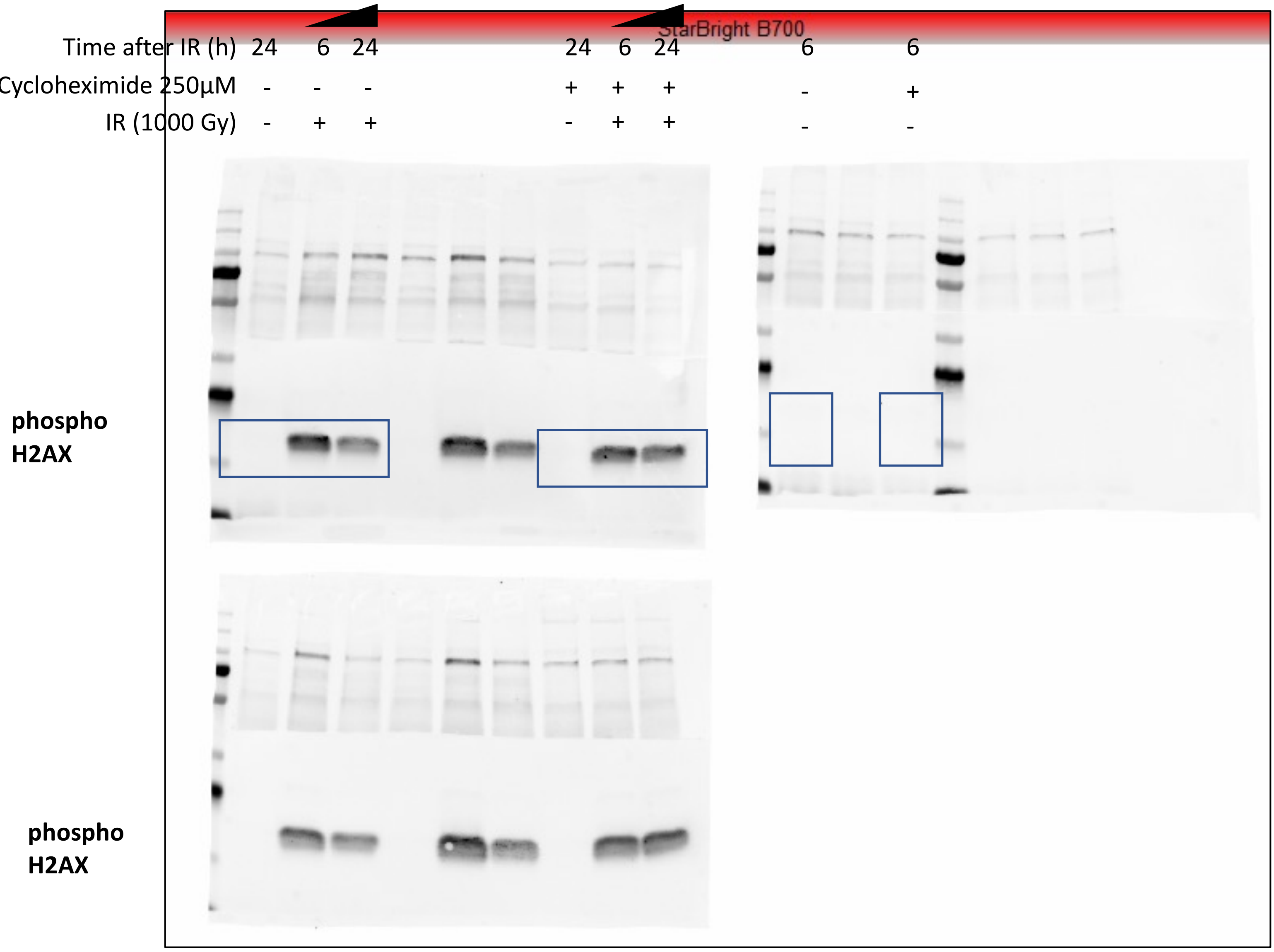
StarBright B700
Time after IR (h) 24 6 24 24 6 24 6 6
Cycloheximide 250µM - - - + + + - +
IR (1000 Gy) - + + - + + - -
phospho H2AX
phospho H2AX
