# Supplementary material for: Comparative transcriptomics reveal a novel tardigrade-specific DNA-binding protein induced in response to ionizing radiation
Source: eLife. 2024 Jul 9;13:RP92621. doi: 10.7554/eLife.92621 (PMC11233135; doi:10.7554/eLife.92621)
Supplement: Supplementary file 5. — (a) g:Profiler analysis of differentially expressed genes in A. antarcticus after IR. (b) g:Profiler analysis of differentially expressed genes in P. fairbanksi after IR. (c) Schematic representation of DNA repair genes up- or downregulated in H. exemplaris after IR. Genes in green colored boxes are upregulated with adjusted p-value<0.05. Genes in red colored boxes are downregulated with adjusted p-value<0.05. Genes with no homolog identified in H. exemplaris genome are checked with a black cross. (d) Table of DNA repair genes up- or downregulated in H. exemplaris, A. antarcticus, or P. fairbanksi after IR, classified based on the KEGG database. Note that the alternative end joining pathway, also called the micro-homology-mediated end joining (MMEJ) pathway is not currently included in the KEGG database. In the KEGG database, the POLQ gene is included in the BER pathway only. Only genes showing differential gene expression with adjusted p-value<0.05 are shown. [file elife-92621-supp5.pdf]

a

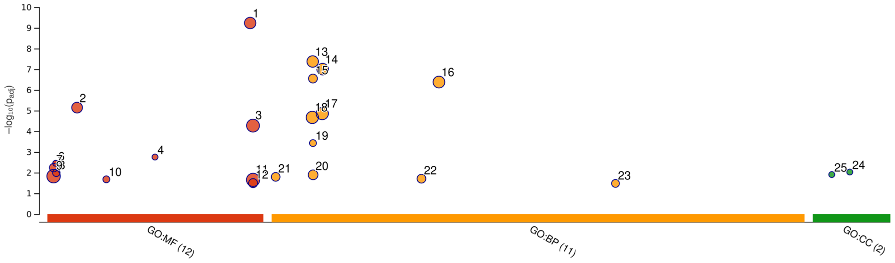

ID	Source	Term ID	Term Name	p_{adj} (query_1)
1	GO:MF	GO:0140097	catalytic activity, acting on DNA	5.827×10^{-10}
2	GO:MF	GO:0008094	ATP-dependent activity, acting on DNA	7.179×10^{-6}
3	GO:MF	GO:0140640	catalytic activity, acting on a nucleic acid	5.338×10^{-5}
4	GO:MF	GO:0042162	telomeric DNA binding	1.768×10^{-3}
5	GO:MF	GO:0003909	DNA ligase activity	3.542×10^{-3}
6	GO:MF	GO:0003910	DNA ligase (ATP) activity	3.542×10^{-3}
7	GO:MF	GO:0003684	damaged DNA binding	5.753×10^{-3}
8	GO:MF	GO:0003950	NAD+ ADP-ribosyltransferase activity	1.023×10^{-2}
9	GO:MF	GO:0003677	DNA binding	1.487×10^{-2}
10	GO:MF	GO:0016886	ligase activity, forming phosphoric ester bonds	2.113×10^{-2}
11	GO:MF	GO:0140657	ATP-dependent activity	2.237×10^{-2}
12	GO:MF	GO:0140658	ATP-dependent chromatin remodeler activity	3.221×10^{-2}
13	GO:BP	GO:0006281	DNA repair	4.234×10^{-8}
14	GO:BP	GO:0006974	DNA damage response	9.943×10^{-8}
15	GO:BP	GO:0006302	double-strand break repair	2.828×10^{-7}
16	GO:BP	GO:0033554	cellular response to stress	4.177×10^{-7}
17	GO:BP	GO:0006950	response to stress	1.399×10^{-5}
18	GO:BP	GO:0006259	DNA metabolic process	2.138×10^{-5}
19	GO:BP	GO:0006303	double-strand break repair via nonhomologous …	3.738×10^{-4}
20	GO:BP	GO:0006310	DNA recombination	1.280×10^{-2}
21	GO:BP	GO:0000723	telomere maintenance	1.604×10^{-2}
22	GO:BP	GO:0032200	telomere organization	1.967×10^{-2}
23	GO:BP	GO:0071897	DNA biosynthetic process	3.324×10^{-2}
24	GO:CC	GO:0043564	Ku70:Ku80 complex	9.374×10^{-3}
25	GO:CC	GO:0030870	Mre11 complex	1.242×10^{-2}

version e109_eg56_p17_1d3191d
date 6/27/2023, 9:55:36 AM
organism hegca002082055v1

g:Profiler

b

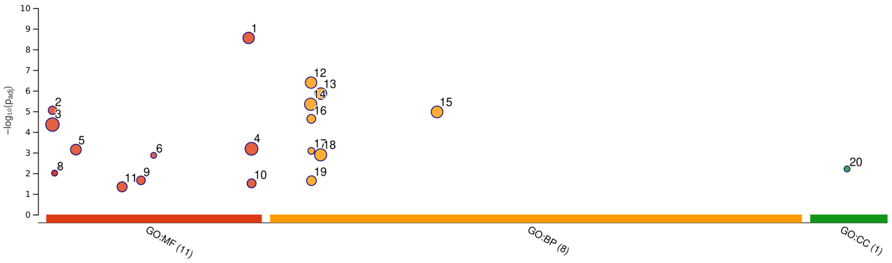

ID	Source	Term ID	Term Name	p_{adj} (query_1)
1	GO:MF	GO:0140097	catalytic activity, acting on DNA	2.786×10^{-9}
2	GO:MF	GO:0003684	damaged DNA binding	8.895×10^{-6}
3	GO:MF	GO:0003677	DNA binding	4.371×10^{-5}
4	GO:MF	GO:0140640	catalytic activity, acting on a nucleic acid	6.492×10^{-4}
5	GO:MF	GO:0008094	ATP-dependent activity, acting on DNA	7.142×10^{-4}
6	GO:MF	GO:0042162	telomeric DNA binding	1.360×10^{-3}
7	GO:MF	GO:0003910	DNA ligase (ATP) activity	9.840×10^{-3}
8	GO:MF	GO:0003909	DNA ligase activity	9.840×10^{-3}
9	GO:MF	GO:0034061	DNA polymerase activity	2.203×10^{-2}
10	GO:MF	GO:0140658	ATP-dependent chromatin remodeler activity	3.083×10^{-2}
11	GO:MF	GO:0030527	structural constituent of chromatin	4.549×10^{-2}
12	GO:BP	GO:0006281	DNA repair	4.044×10^{-7}
13	GO:BP	GO:0006974	DNA damage response	1.389×10^{-6}
14	GO:BP	GO:0006259	DNA metabolic process	4.551×10^{-6}
15	GO:BP	GO:0033554	cellular response to stress	1.069×10^{-5}
16	GO:BP	GO:0006302	double-strand break repair	2.318×10^{-5}
17	GO:BP	GO:0006303	double-strand break repair via nonhomologous …	8.244×10^{-4}
18	GO:BP	GO:0006950	response to stress	1.281×10^{-3}
19	GO:BP	GO:0006310	DNA recombination	2.291×10^{-2}
20	GO:CC	GO:0043564	Ku70:Ku80 complex	6.174×10^{-3}

version e109_eg56_p17_1d3191d
date 6/27/2023, 10:03:30 AM
organism hegca002082055v1

g:Profiler

c

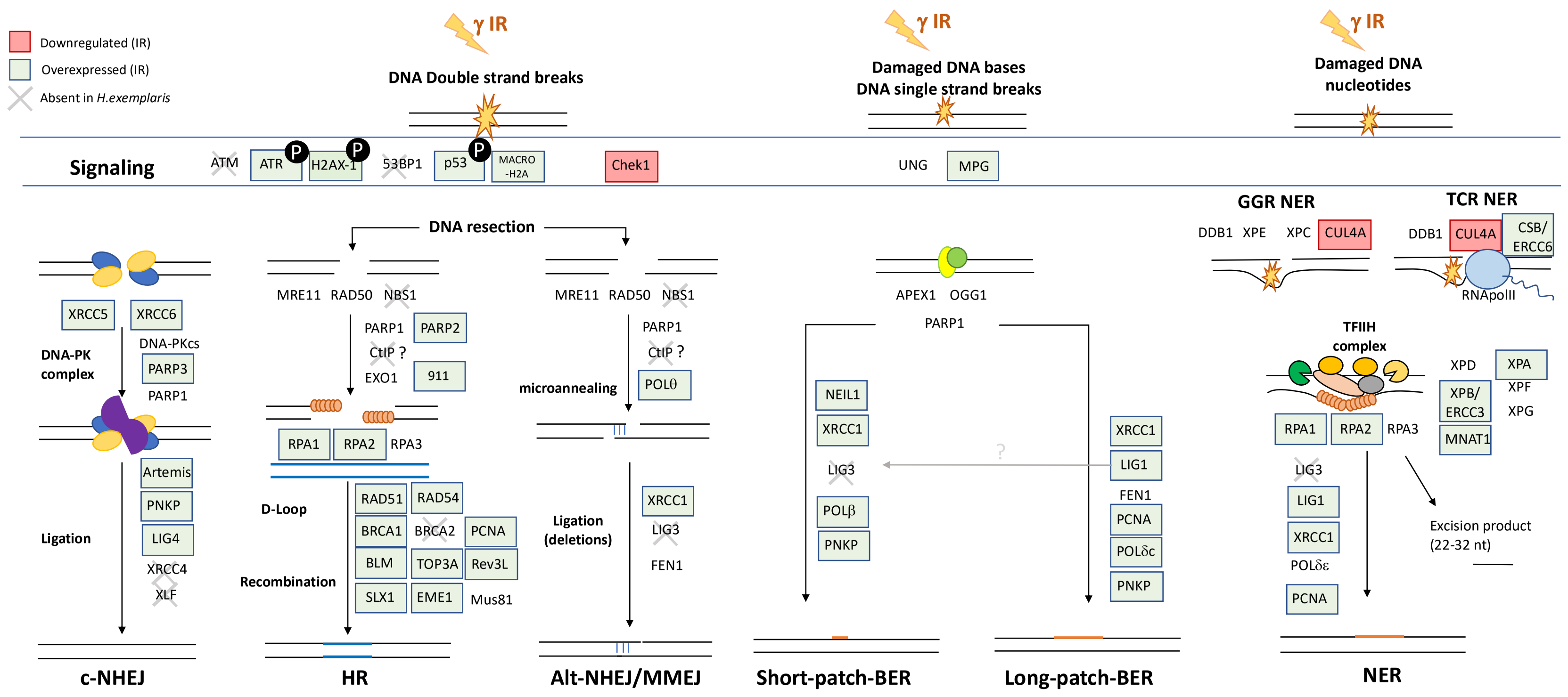
Downregulated (IR)
Overexpressed (IR)
Absent in *H.exemplaris*
γ IR
DNA Double strand breaks
γ IR
Damaged DNA bases
DNA single strand breaks
γ IR
Damaged DNA
nucleotides
Signaling
ATM
ATR
P
H2AX-1
P
53BP1
p53
P
MACRO
-H2A
Chek1
UNG
MPG
GGR NER
DDB1
XPE
XPC
CUL4A
TCR NER
DDB1
CUL4A
CSB/
ERCC6
RNApolII
XRCC5
XRCC6
DNA-PK
complex
DNA-PKcs
PARP3
PARP1
Artemis
PNKP
LIG4
XRCC4
XLF
Ligation
c-NHEJ
DNA resection
MRE11
RAD50
NBS1
PARP1
PARP2
CtIP ?
EXO1
911
RPA1
RPA2
RPA3
D-Loop
Recombination
RAD51
RAD54
BRCA1
BRCA2
PCNA
BLM
TOP3A
Rev3L
SLX1
EME1
Mus81
HR
MRE11
RAD50
NBS1
PARP1
CtIP ?
POLθ
microannealing
Ligation
(deletions)
XRCC1
LIG3
FEN1
Alt-NHEJ/MMEJ
APEX1
OGG1
PARP1
NEIL1
XRCC1
LIG3
POLβ
PNKP
Short-patch-BER
?
XRCC1
LIG1
FEN1
PCNA
POLδc
PNKP
Long-patch-BER
TFIIH
complex
XPD
XPA
XPB/
ERCC3
XPF
XPG
MNAT1
RPA1
RPA2
RPA3
LIG3
LIG1
XRCC1
POLδε
PCNA
Excision product
(22-32 nt)
NER

d

	term_name	Base excision repair			Nucleotide excision repair			Mismatch repair			Homologous recombination			Non-homologous end-joining			Fanconi anemia pathway		
	term_id	KEGG:03410			KEGG:03420			KEGG:03430			KEGG:03440			KEGG:03450			KEGG:03460		
	Species	*H.exemplaris*	*A.antarcticus*	*P.fairbanksi*	*H.exemplaris*	*A.antarcticus*	*P.fairbanksi*	*H.exemplaris*	*A.antarcticus*	*P.fairbanksi*	*H.exemplaris*	*A.antarcticus*	*P.fairbanksi*	*H.exemplaris*	*A.antarcticus*	*P.fairbanksi*	*H.exemplaris*	*A.antarcticus*	*P.fairbanksi*
Differentially expressed genes	APEX1		APEX1														ATR		ATR
	APEX2		APEX2																
	ATR																		
	BLM										BLM	BLM	BLM				BLM	BLM	BLM
	BRIP1										BRIP1		BRIP1				BRIP1		BRIP1
	CCNH				CCNH														
	CDK7				CDK7	CDK7	CDK7												
	CUL4A					CUL4A	CUL4A												
	DCLRE1C													DCLRE1C					
	DDB1				*DDB1*		DDB1												
	ERCC1				ERCC1												ERCC1		
	ERCC2					ERCC2													
	ERCC3				ERCC3	ERCC3	ERCC3												
	ERCC4				ERCC4		ERCC4										ERCC4		ERCC4
	ERCC5						ERCC5												
	ERCC6				ERCC6	ERCC6	ERCC6												
	FAN1																FAN1		
	FANCM																	FANCM	FANCM
	FEN1			FEN1												FEN1			
	GTF2H1				GTF2H1														
	GTF2H2				GTF2H2		GTF2H1												
	GTF2H3					GTF2H3	GTF2H3												
	GTF2H4				GTF2H4		GTF2H4												
	GTF2H5				GTF2H5														
	HES1																		HES1
	HMGB1	HMGB1	HMGB1	HMGB1															
	LIG1	LIG1	LIG1	LIG1	LIG1	LIG1	LIG1	LIG1	LIG1	LIG1									
	LIG4					POLD1								LIG4	LIG4	LIG4			
	MLH1					POLD2				MLH1									MLH1
	MLH3					POLD4				MLH3									
	MNAT1				MNAT1		MNAT1												
	MPG	MPG																	
	MRE11A											MRE11A	MRE11A		MRE11A	MRE11A			
	MSH2								MSH2	MSH2									
	MUS81											MUS81	MUS81					MUS81	MUS81
	NEIL1	NEIL1	NEIL1	NEIL1															
	OGG1		OGG1																
	PARP1			PARP1															
	PARP2	PARP2	PARP2	PARP2															
	PARP3	PARP3	PARP3	PARP3															
	PARP4	PARP4																	
	PCNA	PCNA		PCNA	PCNA		PCNA	PCNA		PCNA									
	POLB			POLB				POLD1	POLD1	POLD1									
	POLD1	POLD1	POLD1	POLD1	POLD1		POLD1	POLD2	POLD2	POLD2	POLD1	POLD1	POLD1						
	POLD2	POLD2	POLD2	POLD2	POLD2		POLD2			POLD3	POLD2	POLD2	POLD2						
	POLD3			POLD3			POLD3	POLD4	POLD4				POLD3						
	POLD4	POLD4	POLD4		POLD4						POLD4	POLD4							
	POLE			POLE			POLE												
	POLE2			POLE2			POLE2												
	POLE4	POLE4			POLE4														
	POLH																	POLH	POLH
	POLK																POLK	POLK	POLK
	POLL	POLL	POLL	POLL										POLL	POLL	POLL			
	PRKDC															PRKDC			
	RAD23A					RAD23A	RAD23A												
	RAD23B				RAD23B		RAD23B								RAD50	RAD50			
	RAD50											RAD50	RAD50						
	RAD51										RAD51	RAD51	*RAD51*				RAD51	RAD51	*RAD51*
	RAD54B											RAD54B							
	RAD54L										RAD54L	RAD54L	RAD54L						
	REV1																REV1	REV1	REV1
	REV3L																REV3L	REV3L	REV3L
	RFC1				RFC1	RFC1	RFC1	RFC1	RFC1	RFC1									
	RFC4						RFC4			RFC4									
	RFC5				RFC5		RFC5	RFC5		RFC5									
	RMI1																RMI1		RMI1
	RPA1				RPA1			RPA1			RPA1						RPA1		
	RPA2				*RPA2*		RPA2	*RPA2*		RPA2	*RPA2*		RPA2				*RPA2*		RPA2
	RPA3						RPA3			RPA3			RPA3						RPA3
	SHFM1												SHFM1						
	SLX4																*SLX4*		SLX4
	SSBP1								SSBP1			SSBP1							
	TOP3A										TOP3A	TOP3A	TOP3A				TOP3A	TOP3A	TOP3A
	TOP3B											TOP3B	TOP3B					TOP3B	TOP3B
	TOPBP1												TOPBP1						
	UNG			UNG															
	XPA				XPA	XPA	XPA												
	XPC				XPC		XPC												
	XRCC1	*XRCC1*	XRCC1	XRCC1															
	XRCC5													XRCC5	XRCC5	XRCC5			
	XRCC6													XRCC6	XRCC6	XRCC6			
