# Supplementary material for: Comparative transcriptomics reveal a novel tardigrade-specific DNA-binding protein induced in response to ionizing radiation
Source: eLife. 2024 Jul 9;13:RP92621. doi: 10.7554/eLife.92621 (PMC11233135; doi:10.7554/eLife.92621)
Supplement: Supplementary file 7. — (a) Scanning electron microscope (SEM) of adult specimen with magnification of mouth and claws. (b) SEM of egg with magnification of characteristic spikes decorating the egg surface. Bright-field morphological analysis performed in parallel by one of the co-authors (R Guidetti) confirmed P. fairbanksi identification. Species identification was further confirmed by 28S, 18S, COX1, ITS2 sequencing (see next page). For further information on P. fairbanksi, see Kayastha et al., 2023. [file elife-92621-supp7.docx]

**a**


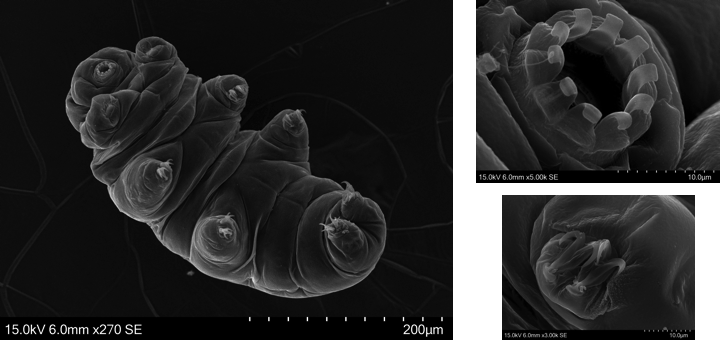


**b**


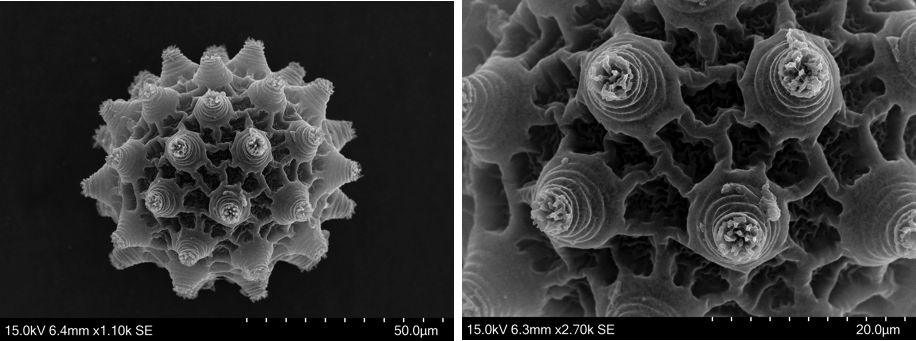


>Pdec_18S

ATCTGATCGGAAACAGAACTGAACGCAGTTCAAGAGCGCTCAGGATTACTTGTAGCGTGCAAGTATATATCCTGACTCGA

TACGTGCTCGTTGGTATGGTATGCATTCTCTCGATCGCTTGACGGGGTATGAATTGCGAGCCATCTGTGCCGCTGCGCCA

GAAGGTAAGCACAAACCGCATATCGCTGTAAACCTGGCCAGCGCAGTGAAGCTGGTGTCTAGGATACTGTCGATGGTTTG

TTCTAACACTCCTACTGCGTCGAAGTTGCAGCAGCGCTTACGCGCTTACCGACCGATGTAGTACGTGGACAGGTACGCGT

TTCTCGCTTGTTATGCGTTGTGTAGCGTGCACAGCGGACAACGATGGAACAGTATCCGATAAAATGTCCAATGATTGCTA

CCTGGTTGATCCTGCCAGTAGTCATATGCTTGTCTCAAAGATTAAGCCATGCATGTCTCAGTACTTGCTATCACAAGGCG

AAACCGCGAATGGCTCATTAAATCAGTTATGGTTCACTAGATCGTACCGTTTACACGGATAACTGTGGTAATTCTAGAGC

TAATACGTGCAACCAGCTCGTTTCCTCGTGAAGCGAGCGCAGTTATTAGAACAAGACCAATCCGGCCTTCGGGTCGGTAA

CTTGGTGACTCTGAATAACCGAAGCAGAGCGCATGGTCTCGTACCGGCGCCAGATCTTTCAAGTGTCTGACTTATCAGCT

TGTTGTTAGGTTATGTTCCTAACAAGGCTATTACGGGTGACGGGGTATCAGGGTCCGATACCGGAGAGGGAGCCTGAGAA

ACGGCTACCACATCCAAGGAAGGCAGCAGGCGCGCAAATTACCCACTCCCAGCACGGGGAGGTAGTGACGAAAAATAACG

ATGCGAGGGCTTTATGCCTCTCGCAATCGGAATGGGTACACTTTAAATCCTTTAACGAGGATCTATTGGAGGGCAAGTCT

GGTGCCAGCAGCCGCGGTAATTCCAGCTCCAATAGCGTATATTAAAGTTGCTGCGGTTAAAAAGCTCGTAGTTGAATCTG

GGCAGTTGGACGGATGGTGCGCTTCACAGCGCTACTGTCTGCTCGGCGCCACAAGCCGGCCATGTCTTGCATGCCCTTCA

CTGGGTGTGCTTGGCGACCGGAACGTTTACTTTGAAAAAATTAGAGTGCTCAAAGCAGGCGTACGGCCTTGCATAATGGT

GCATGGAATAATGGAATAGGACCTCGGTTCTATTTTGTTGGTTTTCGGAACTCGAGGTAATGATTAATAGGAACAGACGG

GGGCATTCGTATTGCGGCGTTAGAGGTGAAATTCTTGGATCGTCGCAAGACGAACTACTGCGAAGGCATTTGCCAAGAAT

GTTTTCATTAATCAAGAACGAAAGTTAGAGGTTCGAAGGCGATCAGATACCGCCCTAGTTCTAACCATAAACGATGCCAA

CCAGCGATCCGTCGGTGTTTATTTGATGACTCGACGGGCAGCTTCCGGGAAACCAAAGTGCTTAGGTTCCGGGGGAAGTA

TGGTTGCAAAGCTGAAACTTAAAGGAATTGACGGAAGGGCACCACCAGGAGTGGAGCCTGCGGCTTAATTTGACTCAACA

CGGGAAAACTTACCCGGCCCGGACACTGTAAGGATTGACAGATTGAGAGCTCTTTCTTGATTCGGTGGGTGGTGGTGCAT

GGCCGTTCTTAGTTGGTGGAGCGATTTGTCTGGTTAATTCCGATAACGAACGAGACTCTAGCCTGCTAAATAGCCAACTG

ATCCGCAGCGTCAGTTGCTACAAAAGCTTCTTAGAGGGACAGGCGGCGTTTAGTCGCACGAGATTGAGCAATAACAGGTC

TGTGATGCCCTTAGATGTCCGGGGCCGCACGCGCGCTACACTGAAGGGAGCAACGTGCTTAATCACCTTGGCCGGAAGGC

CTGGGGAATCCGATTAAACCCCTTCGTGATTGGGATTGAGCTTTGTAATTATCGCTCATGAACGAGGAATTCCCAGTAAG

CGCGAGTCATAAGCTCGCGTTGATTACGTCCCTGCCCTTTGTACACACCGCCCGTCGCTACTACCGATTGAATGATTTAG

TGAGGTCTTCGGACTGGCCATCGAGGCTGCCGCAAGGCGGCTTCGTCTGGTTGGGAAGACGACCAAACTGGCTCATTTAG

AGGAAGTAAAAGTCGTAACAAGGTTTCCGTAGGTGAACCTGCGGAAGGATCATTAACGTGTAATCGGGCGGCGTGTGCCT

GTGCGCTAGGTCTTCGGGCCTGGCCGGGTGCCGTGCTCGTACGCGATAAGCGCTTCTAGCGCTTAACCTGAAAGTCGAGT

CTACACGAACGGTGACTCGCCTATCGTTACTATCCGGAGCGTTTGGTCAGCAATCGCAGCGTGCTTTGCCGACTGCCGGG

GTACGGTAGTGAACTGGTAACTTCGGTACACCGTCGCATGTTGATTTACCTGTCGGAGCGTGAACGGGTGGTCGACACTG

TAATGGCTGTGCCACGGGATACCTACCGTTCTACAAGACGATGCTCTGCGGTCGCTTGAATAACTCACGCGAGACCGTGG

AGAGGCATTCGCCGTAGAATGAGGGCCAGCAGCGCCCAAGGGTATAAAGTAGCTGCAACGAGCGTCGTTGCCGGTTATAT

CCACGATAACGCATCTGTCGTGGGGTATATACCGAATTGGCCGTCCCGCTGCATTTC

>Pdec_ITS2

TCAAAGTGCTTTTCAATTTTCCCTCACGGTACTTGTTCGCTATCGGACTCGTGGTCATATTTAGCCTTCGATGGAGTTTA

CCACCGACTTCACCTTGTACTCACAAACAAGGCGACTCTCCGAAGATGTACACCGGCATCAACTGCTCACGATACGGGCC

TTGCACCCTCCATGGGATGGAGCCTCACTCAGGAGAACTTACGCAAGCAAGCCACACCGGTAACCACCTTCTTCACGCCA

CAGTTCCTGTCACCGTTACCAGCAACAGGATTCGGCGCTGGGCTGTTCCCCTTTCACTCGCAGTTACTATGGGAATCCCC

GTTGGTTTCTTTTCCTCCGCTTAGTAATATGCTTAAGTTCAGCGGGTAATCTCGTCTGAGCTGAGGTCAAAAGATGAGTG

TAGCGAAGCGCAAGTGATTCTTCACGGCCCTGTTCTACAGTTTACTTGCTCCGTAGATTGGGACCTGTTCACCGCTCAAC

GCATAGTTGGCACAGATACGCCACGAACGTGTTCTGTTTTGCTGAACCACTGAAGTGCCGTTGGCAGCTAATCGATACAC

GTGCAACTGCGGCGCACGGCTTCGACAGCCTTCTGCTCCAACTAGCAACACCAGCATTTACCGGTCAGCTAACGAGAACG

CGTATCTGGTCTAGATTTCATCTGATAGCGCCTTATGCAAGCGCTAGCCAGACAATCCGTAGCTTTCACGCAGCTACGAT

TAGTTTTAATCAACTGACCCTCAACCAGGCGTGGCTTCAGTTAACCCGAAGCCGCAATGTGCGTTCGAAGATCCGACGTT

CACAAAGTCCTGCAATTCACGTCGCGTCTCGCATTTTGCTGCGTTCTTCATCGACCCACGAGCCGAATGATCCACCGCAC

AGAGTGATTCATCGGTTTGCATTTTTCGGCTGCTGCTGGGCTGCTGTCTTGCTGACGAGCGCGCCACAACATCAAGCCTC

AAATAGAATACCTTGATACAAATAACGCATTTCCGAAGAAATGCAGCGGGACGGCCAATTCGGTATATACCCCACGACAG

ATGCGTTATCGTGGATATAACCGGCAACGACGCTCGTTGCAGCTACTTTATACCCTTGGGG

>Pdec_COI

CTGCGATGATTTTTCTCTACAAACCACAAAGATATTGGGACTCTCTATTTTATTTTTGGGCTTTGGGCAGCCACCATTGG

GACCTCTTTGAGATTTATTATCCGATCTGAATTAAGCCAACCTGGACAATTGTTTGCAGACGAGCAATTATTTAATGTTA

CAGTAACAAGACATGCCTTTGTTATAATTTTCTTTTTTGTGATACCTATTCTTATTGGGGGGTTTGGTAACTGATTGGTC

CCCCTCATAATTGGGGCTCCAGATATGGCTTTTCCTCGAATAAACAACTTAAGATTTTGACTCCTGCCTCCTTCTTTTCT

TCTTATCCTTATGGGAACAATGGCAGAACAAGGGGCGGGTACTGGATGAACTGTTTACCCGCCACTCTCCCACTATTTTG

CTCATAGCGGCCCTAGGGTTGACCTAACAATTTTTTCTCTTCATATCGCCGGAGCATCTTCTATTTTAGGAGCTATTAAT

TTTATTACTACAATTCTTAATATACGATCTTATTCTATAAGAATAGAGCAAATACCTTTATTTGTATGGTCAGTGCTTAT

CACCGCTATTTTACTCCTTTTAGCTCTACCCGTTTTAGCTGGGGCTATTACTATACTACTTCTAGACCGAAATTTTAATA

CTTCTTTTTTTGACCCAGCAGGAGGGGGGGACCCTATTTTATACCAGCATCTGTTTTGATTCTTTGGCCACCCAGAGGTC

TACATTCTAATTCTTCCGGGATTTGGTATTATTTCTCAAGTTATTATCCACTTTAGAGGAAAGTCACTAACATTTGGACA

TTTGGGTATAATTTATGCAATAAGAACAATCGGCCTATTGGGATTTATTGTGTGAGCACACCATATGTTCACAGTAGGTA

TAGACTTAGATACCCGTGCATACTTTACAGCCGCCACTATAATTATTGCCATTCCTACAGGTGTAAAAGTTTTTAGATGA

CTAAGAACAATTTACGGAAGAAAAATTACATTTAGGGCCCCGATATGATGAGCCCTGGGATTTATTTTCCTTTTTACCCT

GGGAGGACTGACAGGGATTGTGTTATCAAATTCAAGAATTGATATTGCTCTCCATGATACTTACTACGTGGTCGCCCACT

TTCACTACGTCCTGTCTATAGGAGCAGTTTTTGCAATTATTTGCGGGGTAGCTCACTGATTCCCTCTTTTGATAGGGGTT

CAAATGAACAATAAATGACTCCAATCCCAGTTTTTGATTATATTTATTGGGGTGAATATAACCTTTTTCCCTCAACATTT

TCTAGGGTTGGCCGGCATACCACGACGATATGTAGATTACCCAGACACCTTTTTTTCGTGGAACATGGCCTCTTCTTTTG

GGTCCTTATTATCAGCACTCTCTGTTATTTTTCTTTTTTTTATTCTATGAGAAGCAATTGTTTCACAACGTTCAACGTAC

CCGGTGTA

>Pdec_28S

GGATAACTGGTTAATACCAGAACGACGATCAGTACATCTGATCGGAAACAGAACTGAACGCAGTTCAAGAGCGCTCAGGA

TTACTTGTAGCGTGCAAGTATATATCCTGACTCGATACGTGCTCGTTGGTATGGTATGCATTCTCTCGATCGCTTGACGG

GGTATGAATTGCGAGCCATCTGTGCCGCTGCGCCAGAAGGTAAGCACAAACCGCATATCGCTGTAAACCTGGCCAGCGCA

GTGAAGCTGGTGTCTAGGATACTGTCGATGGTTTGTTCTAACACTCCTACTGCGTCGAAGTTGCAGCAGCGCTTACGCGC

TTACCGACCGATGTAGTACGTGGACAGGTACGCGTTTCTCGCTTGTTATGCGTTGTGTAGCGTGCACAGCGGACAACGAT

GGAACAGTATCCGATAAAATGTCCAATGATTGCTACCTGGTTGATCCTGCCAGTAGTCATATGCTTGTCTCAAAGATTAA

GCCATGCATGTCTCAGTACTTGCTATCACAAGGCGAAACCGCGAATGGCTCATTAAATCAGTTATGGTTCACTAGATCGT

ACCGTTTACACGGATAACTGTGGTAATTCTAGAGCTAATACGTGCAACCAGCTCGTTTCCTCGTGAAGCGAGCGCAGTTA

TTAGAACAAGACCAATCCGGCCTTCGGGTCGGTAACTTGGTGACTCTGAATAACCGAAGCAGAGCGCATGGTCTCGTACC

GGCGCCAGATCTTTCAAGTGTCTGACTTATCAGCTTGTTGTTAGGTTATGTTCCTAACAAGGCTATTACGGGTGACGGGG

TATCAGGGTCCGATACCGGAGAGGGAGCCTGAGAAACGGCTACCACATCCAAGGAAGGCAGCAGGCGCGCAAATTACCCA

CTCCCAGCACGGGGAGGTAGTGACGAAAAATAACGATGCGAGGGCTTTATGCCTCTCGCAATCGGAATGGGTACACTTTA

AATCCTTTAACGAGGATCTATTGGAGGGCAAGTCTGGTGCCAGCAGCCGCGGTAATTCCAGCTCCAATAGCGTATATTAA

AGTTGCTGCGGTTAAAAAGCTCGTAGTTGAATCTGGGCAGTTGGACGGATGGTGCGCTTCACAGCGCTACTGTCTGCTCG

GCGCCACAAGCCGGCCATGTCTTGCATGCCCTTCACTGGGTGTGCTTGGCGACCGGAACGTTTACTTTGAAAAAATTAGA

GTGCTCAAAGCAGGCGTACGGCCTTGCATAATGGTGCATGGAATAATGGAATAGGACCTCGGTTCTATTTTGTTGGTTTT

CGGAACTCGAGGTAATGATTAATAGGAACAGACGGGGGCATTCGTATTGCGGCGTTAGAGGTGAAATTCTTGGATCGTCG

CAAGACGAACTACTGCGAAGGCATTTGCCAAGAATGTTTTCATTAATCAAGAACGAAAGTTAGAGGTTCGAAGGCGATCA

GATACCGCCCTAGTTCTAACCATAAACGATGCCAACCAGCGATCCGTCGGTGTTTATTTGATGACTCGACGGGCAGCTTC

CGGGAAACCAAAGTGCTTAGGTTCCGGGGGAAGTATGGTTGCAAAGCTGAAACTTAAAGGAATTGACGGAAGGGCACCAC

CAGGAGTGGAGCCTGCGGCTTAATTTGACTCAACACGGGAAAACTTACCCGGCCCGGACACTGTAAGGATTGACAGATTG

AGAGCTCTTTCTTGATTCGGTGGGTGGTGGTGCATGGCCGTTCTTAGTTGGTGGAGCGATTTGTCTGGTTAATTCCGATA

ACGAACGAGACTCTAGCCTGCTAAATAGCCAACTGATCCGCAGCGTCAGTTGCTACAAAAGCTTCTTAGAGGGACAGGCG

GCGTTTAGTCGCACGAGATTGAGCAATAACAGGTCTGTGATGCCCTTAGATGTCCGGGGCCGCACGCGCGCTACACTGAA

GGGAGCAACGTGCTTAATCACCTTGGCCGGAAGGCCTGGGGAATCCGATTAAACCCCTTCGTGATTGGGATTGAGCTTTG

TAATTATCGCTCATGAACGAGGAATTCCCAGTAAGCGCGAGTCATAAGCTCGCGTTGATTACGTCCCTGCCCTTTGTACA

CACCGCCCGTCGCTACTACCGATTGAATGATTTAGTGAGGTCTTCGGACTGGCCATCGAGGCTGCCGCAAGGCGGCTTCG

TCTGGTTGGGAAGACGACCAAACTGGCTCATTTAGAGGAAGTAAAAGTCGTAACAAGGTTTCCGTAGGTGAACCTGCGGA

AGGATCATTAACGTGTAATCGGGCGGCGTGTGCCTGTGCGCTAGGTCTTCGGGCCTGGCCGGGTGCCGTGCTCGTACGCG

ATAAGCGCTTCTAGCGCTTAACCTGAAAGTCGAGTCTACACGAACGGTGACTCGCCTATCGTTACTATCCGGAGCGTTTG

GTCAGCAATCGCAGCGTGCTTTGCCGACTGCCGGGGTACGGTAGTGAACTGGTAACTTCGGTACACCGTCGCATGTTGAT

TTACCTGTCGGAGCGTGAACGGGTGGTCGACACTGTAATGGCTGTGCCACGGGATACCTACCGTTCTACAAGACGATGCT

CTGCGGTCGCTTGAATAACTCACGCGAGACCGTGGAGAGGCATTCGCCGTAGAATGAGGGCCAGCAGCGCCCAAGGGTAT

AAAGTAGCTGCAACGAGCGTCGTTGCCGGTTATATCCACGATAACGCATCTGTCGTGGGGTATATACCGAATTGGCCGTC

CCGCTGCATTTCTTCGGAAATGCGTTATTTGTATCAAGGTATTCTATTTGAGGCTTGATGTTGTGGCGCGCTCGTCAGCA

AGACAGCAGCCCAGCAGCAGCCGAAAAATGCAAACCGATGAATCACTCTGTGCGGTGGATCATTCGGCTCGTGGGTCGAT

GAAGAACGCAGCAAAATGCGAGACGCGACGTGAATTGCAGGACTTTGTGAACGTCGGATCTTCGAACGCACATTGCGGCT

TCGGGTTAACTGAAGCCACGCCTGGTTGAGGGTCAGTTGATTAAAACTAATCGTAGCTGCGTGAAAGCTACGGATTGTCT

GGCTAGCGCTTGCATAAGGCGCTATCAGATGAAATCTAGACCAGATACGCGTTCTCGTTAGCTGACCGGTAAATGCTGGT

GTTGCTAGTTGGAGCAGAAGGCTGTCGAAGCCGTGCGCCGCAGTTGCACGTGTATCGATTAGCTGCCAACGGCACTTCAG

TGGTTCAGCAAAACAGAACACGTTCGTGGCGTATCTGTGCCAACTATGCGTTGAGCGGTGAACAGGTCCCAATCTACGGA

GCAAGTAAACTGTAGAACAGGGCCGTGAAGAATCACTTGCGCTTCGCTACACTCATCTTTTGACCTCAGCTCAGACGAGA

TTACCCGCTGAACTTAAGCATATTACTAAGCGGAGGAAAAGAAACCAACGGGGATTCCCATAGTAACTGCGAGTGAAAGG

GGAACAGCCCAGCGCCGAATCCTGTTGCTGGTAACGGTGACAGGAACTGTGGCGTGAAGAAGGTGGTTACCGGTGTGGCT

TGCTTGCGTAAGTTCTCCTGAGTGAGGCTCCATCCCATGGAGGGTGCAAGGCCCGTATCGTGAGCAGTTGATGCCGGTGT

ACATCTTCGGAGAGTCGCCTTGTTTGTGAGTACAAGGTGAAGTCGGTGGTAAACTCCATCGAAGGCTAAATATGACCACG

AGTCCGATAGCGAACAAGTACCGTGAGGGAAAATTGAAAAGCACTTTGAAGAGAGAGCGAAACAGTGCGTGAAACCGCTC

AGAGGCAAGCAGATGGGGCCTCGAAGGCAAAGCAGTGAATTCAGCCGGTGTGGTGCGTGGCTGGTTGGTGTTGGGATCGC

AAGACCCTAGCTGATTATGCTCGCGTGCTGGCCGGTGCATTTTCGCTGTTTGTACGTCACCGCCGTTGAGCGAGCATCCG

TCGGGTATGCGTGTGAAGCCTTATTCCTTCGGGCGTAGGTGCTTACTGCTAACTTGTACGCGTTTGCGCCTCAACTGGTC

ATGTCAGCGTGTGCCAGCGTTAGCGTTGGGCCGGTATGCTCTGCGGTGTGTTGTGGGATGACGAGCTTGCTCGGCTCCTC

GATACGCAGTGGACTCGTTGCCGGTTTTCAACGTAGGCACATTGTAGATTCGGTGGCGAGTAGACGGCTGCCCATCTAAC

CCGTCTTGWAACACGGACCAAGGAGTTCAACATGCGCGCTAGTTGTTGGGACTTGAAGCCCGCTAGCAAAGTGAAAGCAA

GACACAGTGTACGCTGTGTATTGGCGAGATCCCGTCACCTGGTTTACCAGGCCGGGCGCACCGCCGGCCCGTCAAAAGCT

CATGTGGCTTTGGCGGAGCTTGAGCGTGCACGTTGAGACCCGAAAGATGGTGAACTATGCCTGGGCAGGATGAAGCCAGG

GGAAACTCTGGTGGAGGTCCGTAGCGATTCTGACGTGCAAATCGATCGTCTGACCTGGGTATAGGGGCGAAAGACTAATC

GAACCATCTAGTAGCTGGTTCCCTCCGAAGTTTCCCTCAGGATAGCTGGCACTCGAGAGAACGTAGTCTCTCCCGGTAAA

GCGAATGATTAGTGGCCTTGGGGTCGAAACGACCTTAACCAATTCTCAAACTTTAAATGGGTGAGAAGTCCGGCTTGCTT

AAATGCATAGCTGAAGTCCGGACGTTGGATACGAGCGCCTAGTGGGCCACTTTTGGTAAGCAGAACTGGCGCTGTGGGAT

GAACCGAACGCTGAGTTATGGCGCCCGACGAGACGCTCATCAGATCCCAGAAAAGGTGTTGGTTGCTATAGACAGCAGGA

CGGTGGCCATGGAAGTCGGAACCCGCTAAGGAGTGTGTAACAACTCACCTGCCGAAGCAACTAGCCCTGAAAATGGATGG

CGCTAGAGCGTCTGGCCTATACTCGGCCGTTGCAGCAGCAGCAACGTTAAGTCAAGCTGCAACGAGTAG
